# Epigenetic aging signatures and age prediction in human skeletal muscle

**DOI:** 10.18632/aging.206341

**Published:** 2025-11-26

**Authors:** Soo-Bin Yang, Jeong Min Lee, Moon-Young Kim, Soong Deok Lee, Hwan Young Lee

**Affiliations:** 1 Department of Forensic Medicine, Seoul National University College of Medicine, Seoul, Korea; 2 Institute of Forensic and Anthropological Science, Seoul National University College of Medicine, Seoul, Korea; 3 Department of Anatomy and Cell Biology, Laboratory of Forensic Medicine, Sungkyunkwan University School of Medicine, Suwon, Korea

**Keywords:** skeletal muscle, age, DNA methylation, next generation sequencing, single base extension

## Abstract

Aging causes progressive molecular and cellular changes that impair skeletal muscle function. DNA methylation is a key epigenetic regulator of this process, but its role in skeletal muscle, especially in Asian populations and postmortem samples, remains underexplored. We analyzed DNA methylation profiles from 103 pectoralis major muscle samples from autopsies of South Korean individuals (18–85 years) using the Infinium EPIC array. Targeted validation and age prediction modeling were performed with Next-Generation Sequencing (NGS) and Single Base Extension (SBE). We identified 20 age-associated CpG markers linked to genes involved in muscle structure, metabolism, and stress response. Machine learning models built on these CpG sites showed high prediction accuracy, with mean absolute errors of 5.537 years in sequencing and 3.797 years in extension platforms, and strong correlation with chronological age.

This study introduces the skeletal muscle epigenetic clocks in an Asian population using postmortem skeletal muscle tissue. These novel prediction models, based on 20 common CpG markers using SBE and NGS platforms, provide a robust framework for forensic applications and enable population-tailored epigenetic profiling. Beyond predictive utility, the identified age-associated methylation signatures offer valuable insights into the molecular pathways of muscle aging and hold promise as biomarkers for translational research and age-related clinical interventions.

## INTRODUCTION

Aging is a multifaceted biological phenomenon characterized by a progressive decline in cellular function and regenerative capacity, which ultimately contributes to the onset of age-related diseases and a reduction in overall physiological resilience [1, 2]. Of the many biomarkers indicative of the aging trajectory, DNA methylation has been recognized as a notably robust epigenetic marker [3]. These methylation patterns, which influence gene expression without altering the DNA sequence itself, are shaped by both genetic predispositions and environmental factors, providing a nuanced molecular profile of biological aging that extends beyond chronological age alone [4, 5].

The potential to estimate age through DNA methylation patterns has led to significant attention, resulting in the development of several epigenetic clocks, including Horvath’s pan-tissue clock [6], the skin and blood clock [7], GrimAge [8], Levine’s PhenoAge [9] and Voisin’s Muscle Epigenetic Age Test (MEAT) [10, 11]. These epigenetic clocks serve as robust tools for estimating biological or chronological age, with each model specifically designed for either a single tissue type or multiple tissues. Although these clocks have demonstrated notable utility, their predictive performance typically declines when used outside the tissue context for which they were developed. For example, Horvath’s pan-tissue clock exhibited poor calibration in histologically muscular tissues, such as breast, uterine endometrium, skeletal muscle, and heart, underscoring the tissue specificity of DNA methylation profiles [6]. Therefore, biological age, reflecting molecular and physiological changes, does not always coincide with chronological age, which underscores the need for tissue- and context-specific epigenetic clocks.

In particular, skeletal muscle presents distinct challenges for epigenetic age prediction because of its intricate cellular composition and its vital roles in mobility, metabolism, and health across the lifespan [12]. The cellular heterogeneity of skeletal muscle, encompassing myofibers, satellite cells, and various supportive cells, contributes to distinct DNA methylation profiles that differ from those of other tissues [13, 14]. Zykovich’s group identified age-related methylation changes in skeletal muscle from young and older Canadian-Americans, providing insights into muscle-specific aging [15]. To improve age prediction, Voisin’s team established the MEAT clock, an epigenetic clock specifically for skeletal muscle, which enhanced age estimation accuracy in muscle tissue [10, 11]. More recently, proteomic clocks have demonstrated good performance in muscle as well as in various other tissues [16–18]. However, previous studies mostly relied on biopsy samples from limited regions, mainly the vastus lateralis (VL) muscle, and could not fully capture the methylation patterns of different muscle fiber types [19]. Moreover, previous studies have primarily focused on individuals of European ancestry, limiting their applicability to other populations, like Asians, and post-mortem contexts. Given the influence of genetics and environmental factors on epigenetics, further research involving diverse populations and a broader range of anatomical muscle regions is needed to improve muscle-specific epigenetic age models [20].

In this study, we aimed to address current limitations by utilizing pectoralis major (PM) muscle tissue, allowing us to analyze a wider range of muscle tissues from different anatomical locations. This approach helps capture regional variability in DNA methylation patterns within skeletal muscle. Additionally, by focusing on an Asian population, specifically Koreans, we sought to improve the accuracy and applicability of muscle-specific epigenetic clocks, bridging gaps in population diversity overlooked by previous research. This study aimed to identify skeletal muscle–specific CpG markers for age prediction and to develop robust CpG-based epigenetic models. In addition to constructing a novel prediction model from selected CpG methylation sites, we evaluated its robustness under varying DNA input conditions and examined the mRNA expression of genes associated with these CpGs to assess their biological relevance. To further establish tissue specificity, we compared DNA methylation patterns and model performance across skeletal, cardiac, and smooth muscle tissues. Our findings underscore the importance of tissue-specific DNA methylation in age estimation and support the development of practical tools for research and forensic use.

## RESULTS

### Discovery of aging-associated CpG markers from skeletal muscle

**Figure 1:**
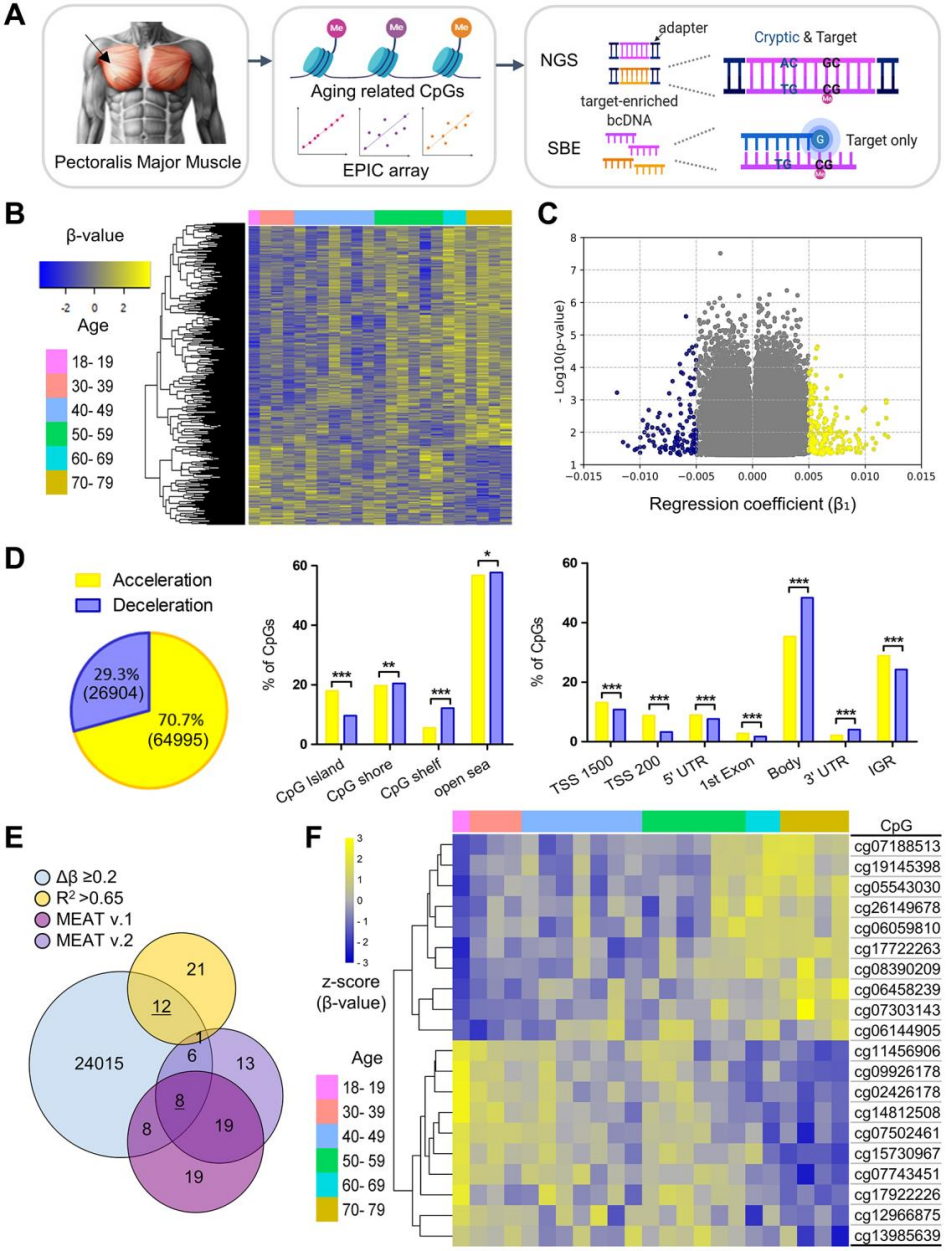
**Identification and characterization of aging-related skeletal muscle-specific CpGs.** (**A**) Schematic overview of experimental workflow. The diagram, created using BioRender, illustrates the workflow starting with the collection of PM muscle tissue, indicated an arrow pointing to the anatomical site clearly demarcated through shaded surrounding areas. DNA was bisulfite-converted and analyzed using the Infinium Methylation EPIC array to identify age-associated CpGs. Cryptic and target CpGs were examined via NGS, while only target CpGs were used for SBE. (**B**) Two-way hierarchical clustering heatmap of methylation profiles from 23 male PM muscle samples profiled by the EPIC array. Samples are color-coded by age group. The heatmap displays 500 randomly selected CpGs from 91,899 significant age-related CpGs (*p* < 0.05), with beta values normalized to Z-scores (yellow for higher, blue for lower values). (**C**) Volcano plot showing regression coefficients (β_1_) from age-related linear regression. Each dot represents a CpG with *p* < 0.05. Yellow and blue dots indicate CpGs with methylation gain and loss with age, respectively. (**D**) Enrichment of significant CpGs by genomic context. The Venn diagram shows the distribution of methylation accelerated (yellow) and decelerated (blue) CpGs among 91,899 significant CpGs. Bar plots display CpG distributions relative to CpG islands (left bar graph) and chromatin regions (right bar graph) using raw counts (the number of CpG sites) and the Chi-square (χ^2^) test. Statistical significance is denoted as ^*^p < 0.05, ^**^p < 0.01 and ^**^p < 0.001. (**E**) Filtering of CpGs through multi-step criteria. The Venn diagram displays the overlap of CpGs filtered by effect size (Δβ ≥ 0.2), model fit (R^2^ > 0.65), and prior muscle-specific clocks (MEAT v.1 and v.2). Twelve and eight final CpGs (underlined) were selected for NGS- and SBE-based modeling. (**F**) Heatmap of the final 20 CpGs selected for age prediction, grouped by age. Beta values are Z-score normalized; age groups are color-coded.

Among the 103 PM muscle samples, 23 male samples aged 18 and 35–78 years were examined using the EPIC array to identify aging-associated CpG markers for Next Generation Sequencing (NGS) and Single Base Extension (SBE)-based model construction (Figure 1A and Supplementary Figure 1). Probes with detection *p*-value greater than 0.05 were excluded, leaving a total of 91,899 high-quality CpG probes for analysis (Supplementary Table 1). Of these, 105 CpG sites met the threshold of an FDR-adjusted *p*-value < 0.1. Initially, a two-way hierarchical clustering heatmap of 500 randomly selected CpGs based on linear regression was generated, revealing distinct age-related methylation patterns (Figure 1B). In particular, among CpGs with a regression coefficient (β_1_) > 0.005, accelerated sites were 1.5 times more prevalent than decelerated ones (181 vs. 122) (Figure 1C). Overall, 70.7% (64,995 CpGs) showed age-related methylation acceleration, while 29.3% (26,904 CpGs) showed deceleration, which aligns with previous findings [21]. Next, enrichment analysis based on CpG island and genomic position was performed using the Chi-square test. Examination of probe counts revealed that accelerated CpGs were widespread across genomic regions classified by CpG density and transcriptional activity, compared to decelerated sites (Figure 1D). Regarding proportion, 30.8% of accelerated CpGs and 21.7% of decelerated CpGs were identified within transcriptional regulatory sites, such as the transcription start site (TSS), including TSS1500 and TSS200, as well as the 5′ untranslated region (UTR) (Figure 1D).

Among the 91,899 filtered CpGs, 12 CpGs were selected as candidate age-associated markers according to the criteria of a maximum to minimum beta value difference (Δβ) > 0.2, R^2^ > 0.65 from linear regression between age and beta value, and an FDR-adjusted *p*-value < 0.08 (Figure 1E and Table 1). To improve marker diversity and generalizability, eight additional CpGs overlapping with MEAT v.1 and v.2 were selected from 24,050 sites with Δβ > 0.2, despite R^2^ < 0.65, enhancing applicability across various populations and muscle types (Figure 1E and Table 1). The final set of 20 muscle-specific CpGs, labeled MA_01 to MA_20, represents the Muscle Age numbering (Table 1). Of these, 10 markers displayed age-related methylation acceleration, while the remaining 10 showed deceleration as determined from the EPIC array data (Figure 1F). We compared the 20 markers with probes from GEO datasets to determine if they were previously reported or novel. Methylation data from these markers were compared with GSE50498, which contains VL muscle samples from 24 young and 24 elderly individuals [15], and GSE114763, consisting of VL muscle samples from 8 Europeans [22] (Supplementary Figure 2). Nine markers showed similar methylation patterns, with R^2^ differences below 0.4 between VL muscle data from Caucasian donors and PM muscle data from Korean samples. The remaining markers, however, showed marked discrepancies. These results suggest that while methylation patterns are generally consistent, differences in predictive accuracy may reflect anatomical and population variations. This highlights the need for new, robust age-related CpG markers and a better understanding of their genomic context.

**Table 1:** Age-related genetic, epigenetic, and transcriptomic changes at skeletal muscle-specific CpG markers.

**Marker**	**CpG_ID**	**Genomic**			**Epigenetic**				**Transcriptomic**		
		**RefGene**	**CpG_Island**	**Position**	**β changes**	**R²**	**Δβ**	***p*-value**	**mRNA Exp.**	***p*-value**	**References**
MA_01	cg06458239	*ZNF549*	Island	TSS200	+	0.702	0.291	6.0E-07	+	0.035	[22, 23]
MA_02	cg02426178	*SLC44A2*	Island	Body	−	0.696	0.218	7.4E-07	+	0.033	[21]
MA_03	cg11456906	*CFAP74*		Body	−	0.688	0.231	1.0E-06	+	0.244	
MA_04	cg07743451	*TPM3*	N_Shelf	Body	−	0.686	0.305	1.1E-06	+	0.011	
MA_05	cg14812508	*TWF2*		Body	−	0.676	0.276	1.5E-06	−	0.011	[21, 22]
MA_06	cg07188513	*MNX1-AS1*	N_Shelf	Body	+	0.672	0.235	1.7E-06	+	0.046	
MA_07	cg07502461	*TWF2*		Body	−	0.667	0.327	2.0E-06	−	0.011	
MA_08	cg09926178	*ACTA1*	Island	5'UTR	−	0.658	0.227	2.6E-06	−	0.004	[22]
MA_09	cg15730967	*CAND2*	Island	Body	−	0.658	0.418	2.7E-06	−	0.869	[22]
MA_10	cg07303143	*KIF15*	Island	Body	+	0.656	0.228	2.8E-06	+	0.019	
MA_11	cg17722263	*MYCNUT*		TSS200	+	0.653	0.214	3.1E-06	+	0.037	
MA_12	cg05543030				+	0.651	0.294	3.3E-06	.	.	
MA_13	cg08390209	*CDKN2B*	N_Shore	3'UTR	+	0.613	0.321	1.0E-05	+	0.063	[22, 23]
MA_14	cg19145398	*FOXS1*	S_Shore	TSS1500	+	0.426	0.223	7.4E-04	+	0.008	
MA_15	cg26149678	*IL18BP*		TSS200	+	0.342	0.230	3.4E-03	+	0.001	[22]
MA_16	cg12966875	*SLPI*		TSS1500	−	0.337	0.269	3.7E-03	+	0.025	[22]
MA_17	cg06059810	*RUFY3*		Body	+	0.320	0.268	4.9E-03	+	0.001	
MA_18	cg17922226	*CLCN1*		Body	−	0.304	0.291	6.4E-03	+	0.000	
MA_19	cg06144905	*PIPOX*		TSS200	+	0.287	0.233	8.5E-03	+	0.004	
MA_20	cg13985639	*HOGA1 (DHDPSL)*		Body	−	0.279	0.241	9.6E-03	+	0.011	

Genomic information was obtained from the Illumina EPIC array manifest, aligned with data from the UCSC Genome Browser. Methylation changes were derived from EPIC array analyses, with age-related regression results presented as statistical indicators with R-squared (R^2^) values and the difference between maximum and minimum beta value (Δβ). Methylation acceleration with aging is indicated by + sign, while methylation deceleration is indicated by - sign. mRNA expression changes between young and old groups, identified in this study, align with trends reported in previous research, highlighting consistent age-associated alterations. An increase in mRNA expression levels in the older group is denoted by + sign, while a decrease is denoted by - sign.

### Genomic analysis and functional implications of the 20 aging-associated CpG markers

Among the target 20 CpGs, 10 markers exhibited methylation acceleration. Nine of these were mapped to genes including *ZNF549, MNX1-AS1, KIF15, MYCNUT, CDKN2B, FOXS1, IL18BP, RUFY3*, and *PIPOX* (Table 1), while one marker (MA_12) was not associated with any annotated gene. Conversely, the 10 CpGs showing methylation deceleration were linked to nine genes including *SLC44A2, CFAP74, TPM3, TWF2, ACTA1, CAND2, SLPI, CLCN1*, and *DHDPSL.* The biological functions and age-associated disease relevance of these differentially methylated genes were evaluated using the Ingenuity Pathway Analysis (IPA) platform to reveal distinct biological pathways influenced by methylation changes with aging.

**Figure 2:**
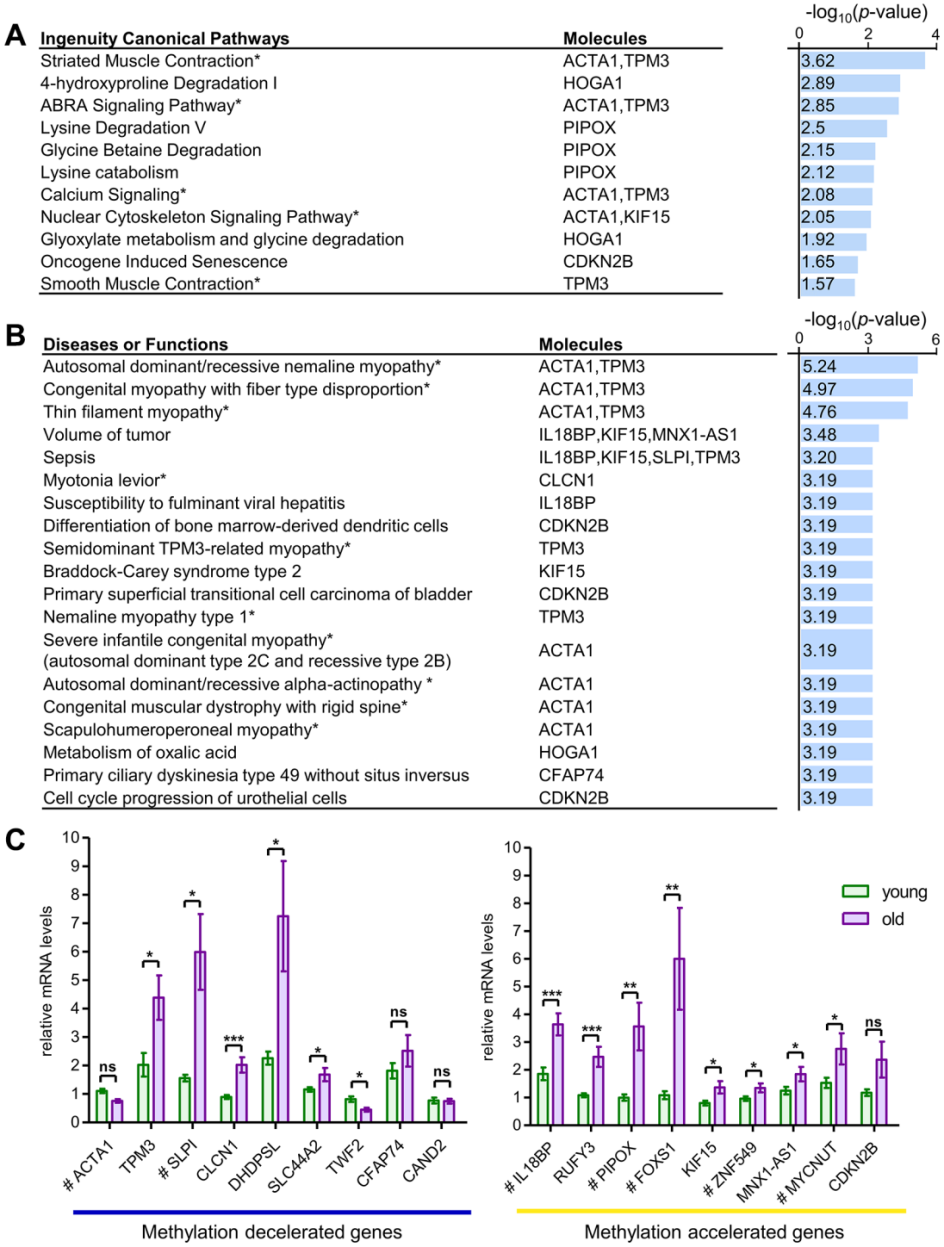
**Functional enrichment and transcriptional analysis of aging-associated skeletal muscle-specific CpG loci.** (**A**, **B**) Ingenuity canonical pathways of aging-related CpGs and classification of diseases and functions. Canonical pathways are ranked by -log(*p*-value) (right x-axis) and associated molecules shown (**A**). Disease and function categories are similarly ranked and visualized (**B**). Pathways directly linked to muscle functions are marked with an asterisk (*). These pathways were identified through IPA program. (**C**) mRNA Transcriptional Levels of Genes Associated with Aging-Related CpGs. Nine genes showed methylation deceleration at the target CpG sites with aging, while nine others displayed methylation acceleration. mRNA transcription levels of the methylation-decelerated genes are presented in the left graph, and the methylation-accelerated genes are shown in the right graph. All mRNA levels correspond to genes with muscle-specific CpGs associated with aging, measured using RT-qPCR in pectoralis major muscle. Tissue samples from 11 individuals were used and categorized into two age groups. The young group (*n* = 6) consisted of individuals aged 21, 24, 27, 29, 33, and 35 years, including three males and three females. The old group (*n* = 5) included individuals aged 63, 65, 68, 74, and 77 years, comprising four males and one female. Genes with target CpGs located in the regulatory regions TSS1500, TSS200, and 5'UTR are marked with a hash (#). Statistical significance was assessed using Student’s *t*-test, with significant differences indicated as ^*^p < 0.05, ^**^p < 0.01, and ^***^p < 0.001. Data are presented as the mean ± standard error of the mean (SEM), highlighting significant differences across groups. IGR, intergenic region.

Functional analysis identified statistically significant differences across 21 canonical pathways (*p* < 0.05), with the top eight pathways (*p* < 0.01) predominantly influenced by five key molecules: *ACTA1, TPM3, HOGA1, PIPOX*, and *KIF15* (Figure 2A). These molecules were linked to muscle functions, contraction, and metabolism. Furthermore, 276 diseases associated with transcriptional changes driven by differential methylation (*p* < 0.05) were identified, with 23 showing stronger significance (*p* < 0.001), including 13 directly related to myopathy (Figure 2B). Notably, three muscle-specific genes, *ACTA1, TPM3*, and *CLCN1*, were associated with hereditary skeletal myopathy and congenital myopathy.

To examine how methylation affects transcription, RNA expression of the 18 genes associated with the 20 target CpGs were analyzed using RT-qPCR (Figure 2C). Significant differences between young and old individuals were observed for 14 of these genes. In particular, expression of the *CLCN1, IL18BP*, and *RUFY3* genes exhibited more than two-fold changes, with highly significant statistical differences (*p* < 0.001). Notably, with advancing aging, mRNA levels of genes exhibiting methylation change in regulatory regions, including the 5′UTR and TSS positions, displayed a positive correlation, except for the *SLPI* gene (Table 1). This trend is potentially explained by DNA methylation occurring within regulatory regions characterized by low CpG density [23]. The RNA expression findings for these 20 genes are consistent with previous transcriptome and proteome investigations [24–26] (Table 1).

### Construction and validation of age prediction model using NGS data

To develop an age prediction model representative of muscle aging, we analyzed NGS data from 20 selected CpG markers. MiSeq sequencing generated approximately 39 million reads that passed the initial quality assessment, with an average of 249,133 read pairs per sample, ranging from 95,134 to 608,471. Following processing with CLC software, the average read count per sample was reduced to 50,892, ranging from 18,043 to 130,532 (Supplementary Figure 3A). Among the 20 target amplicons, 14 demonstrated average coverages between 1,276 and 8,967 reads per CpG, exceeding the 1,000 read threshold for reliable methylation quantification (Supplementary Figure 3B). Although the other six amplicons satisfied the theoretical coverage required for a 90% confidence interval [27], four amplicons with coverage below 700 reads per CpG were excluded from model construction to maintain data reliability.

**Figure 3:**
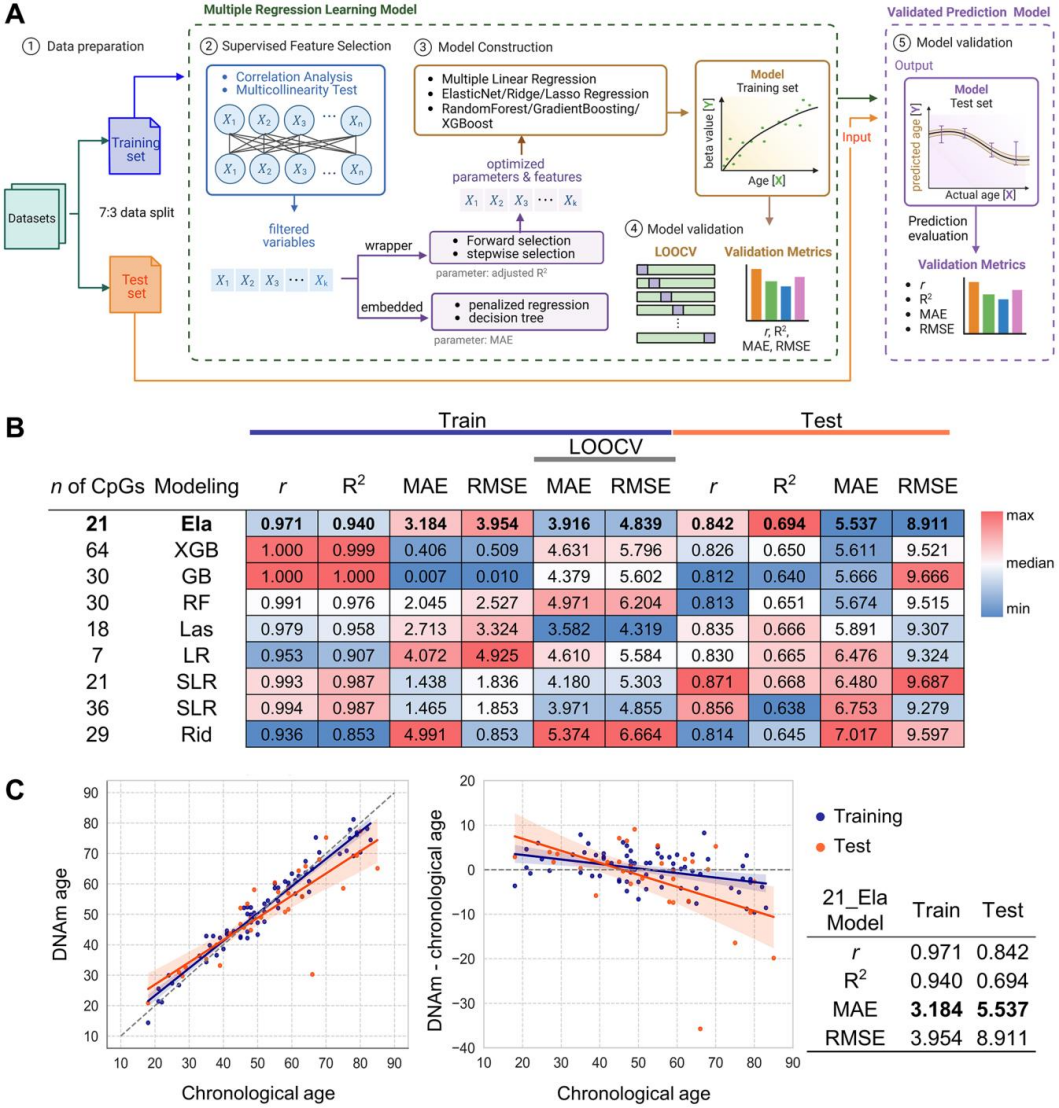
**Development and validation of age prediction models using the NGS system.** (**A**) Schematic of the data processing workflow for NGS and SBE-based models, created using BioRender. Data were divided into training and test sets at a 7:3 ratio. Supervised feature selection involved correlation analysis and multicollinearity filtering, followed by model construction using forward selection, stepwise selection, penalized regression, and decision tree models with optimized parameters. Model validation was performed through LOOCV, with performance evaluated by Pearson’s r, R^2^, MAE, and RMSE. Finally, the validated prediction model was applied to test sets. (**B**) Performance heatmap of nine machine learning models developed from NGS data. Models differ by algorithm (LR, SLR, Ela, Las, Rid, RF, GB, XGB) and CpG set. Metrics (*r*, R^2^, MAE, RMSE) were calculated for both training and test sets (*n* = 103). LOOCV was used for training validation. Heatmap color scale reflects relative performance for each column criterion and the top NGS model is indicated in bold. (**C**) Prediction accuracy of the best-performing NGS model. The best model’s accuracy was evaluated by comparing predicted age with chronological age for 103 samples in both the training (orange) and test (blue) sets (left plot). Residuals between DNA methylation (DNAm) age and chronological age are plotted (right plot). Regression lines are shown with each color, with 95% confidence intervals shaded. Model performance metrics are summarized in the accompanying table. Abbreviations: LOOCV: Leave one out cross validation; LR: Linear regression; SLR: Stepwise Linear Regression; Ela: ElasticNet; Las: Lasso regression; Rid: Ridge regression; XGB: XGBoost; RF: RandomForest; GB: GradientBoosting.

An overview of the machine learning workflow based on NGS and SBE data is illustrated in Figure 3A. In addition to the 16 primary CpGs from the EPIC array, adjacent cryptic CpGs were detected and considered as supplementary candidate loci for model development, potentially capturing subtle but informative age-associated methylation changes (Figure 1A). Model construction included all 16 amplicons, comprising the 16 target CpGs and 54 additional cryptic CpGs. A total of nine models were constructed using various machine learning approaches. Each model was fitted using a training dataset (*n* = 71) and evaluated on an independent test set (*n* = 32), resulting in mean absolute error (MAE) values ranging from 5.537 to 7.017 in the test set (Figure 3B). The detailed formulas of all models are presented in Supplementary Table 2. Of these, the Elastic-net (Ela) model, optimized with an alpha of 0.0178 and an L1 ratio of 0.9852, selected 9 target CpGs and 12 cryptic CpGs across 12 amplicons (Table 2). This model achieved a MAE of 3.184 in the training set and 5.537 in the test set. Although the performance was robust, the discrepancy between training and test MAE implies a risk of overfitting. To address this, we attempted to reduce overfitting by applying stronger regularization and post hoc feature selection, however, these approaches did not improve model performance (Supplementary Table 3). To further evaluate reliability, Leave-One-Out Cross-Validation (LOOCV) was performed on the training set, yielding a MAE of 3.916. Additionally, we performed 5-fold and 10-fold cross-validation, which resulted in MAE values of 3.8629 and 3.8743, respectively. To further assess robustness and determine whether the Ela model captures true age-associated signals rather than random noise, we performed permutation testing, which demonstrated that the model is not simply overfitting to random structure (Supplementary Figure 4). The Elastic Net model was identified as the best-performing approach, exhibiting the lowest MAE in the test set and reduced overfitting compared with other models. Remarkably, the Ela model achieved strong predictive accuracy using only 21 CpGs across 11 amplicons, highlighting the efficiency of a compact marker panel. Collectively, these results establish the Ela model with 21 CpGs as a practical and robust tool for predicting chronological age (Figure 3C and Table 2).

**Table 2:** Regression coefficients of best-performing SBE and NGS-based age prediction models.

**Marker**	**Amplicon**	**Chr:Position**	**SBE coefficient**	**NGS coefficient**
Intercept	.	.	25.31137334	105.9722
MA_01	target	chr19:57527206	87.9153	30.6368
	cryptic	chr19:57527208	.	8.2441
	cryptic	chr19:57527211	.	15.3597
	cryptic	chr19:57527218	.	24.8859
MA_02	target	chr19:10636467	.	−24.7511
	cryptic	chr19:10636497	.	7.3709
MA_03	cryptic	chr1:1937587	.	10.4319
MA_04	target	chr1:154179209	8.7945	−3.3927
MA_08	target	chr1:229433354	−40.7868	0.0001
	cryptic	chr1:229433453	.	12.7462
MA_09	cryptic	chr3:12815282	.	−29.4345
	cryptic	chr3:12815338	.	−11.4447
	cryptic	chr3:12815426	.	−40.0636
	cryptic	chr3:12815466	.	−30.3562
MA_10	cryptic	chr3:44761956	.	9.3494
	target	chr3:44761961	132.3527	53.4929
	cryptic	chr3:44761965	.	29.5532
MA_11	target	chr2:15920273	.	14.5369
MA_13	target	chr9:22005565	28.4466	17.2336
MA_18	target	chr7:143316521	−56.2316	−23.7932
MA_19	target	chr17:29042762	48.0441	38.6166

This table presents the regression coefficients for the CpG markers used in the best-performing age prediction models: the SBE-based 7CpGs_SLR model and the NGS-based 21CpGs_Ela model. The amplicon column indicates whether the CpG marker was part of the skeletal muscle specific CpGs discovered from EPIC array (target) or was additionally identified within the same amplicon using NGS (cryptic). The Chr:Position column shows the chromosomal location of each CpG marker. The coefficients in the SBE and NGS columns represent the contribution of each marker to the final age estimation formula.

### Construction and validation of age prediction model using multiplex SBE data

To ensure practical applicability, we aimed to develop a multiplex SBE system. Initially, we generated methylation data from 68 samples using four multiplexed sets on the SBE platform (Supplementary Figure 5). The association between age and methylation levels at each marker was evaluated using Pearson’s r from linear regression analysis. Among the 20 target CpG markers identified in this study, 3 demonstrated strong correlations (r > 0.7), including MA_05 (r = 0.7488), MA_10 (r = 0.8174), and MA_19 (r = 0.7865), and 12 exhibited moderate correlations (0.5 < r < 0.7) (Supplementary Figure 6). Before constructing predictive models, the methylation data were evaluated for adherence to statistical assumptions and normality. Values for skewness (−0.059) and kurtosis (−0.476) indicated a normal distribution (Supplementary Figure 7A). Normality of residuals was confirmed through a Q-Q plot, and the Durbin-Watson test (1.684) supported the independence of errors without autocorrelation (Supplementary Figure 7B). Through the multicollinearity test, marker MA_14 was excluded due to a variance inflation factor of 11.25, exceeding the threshold of 10 (Supplementary Figure 7C).

**Figure 4:**
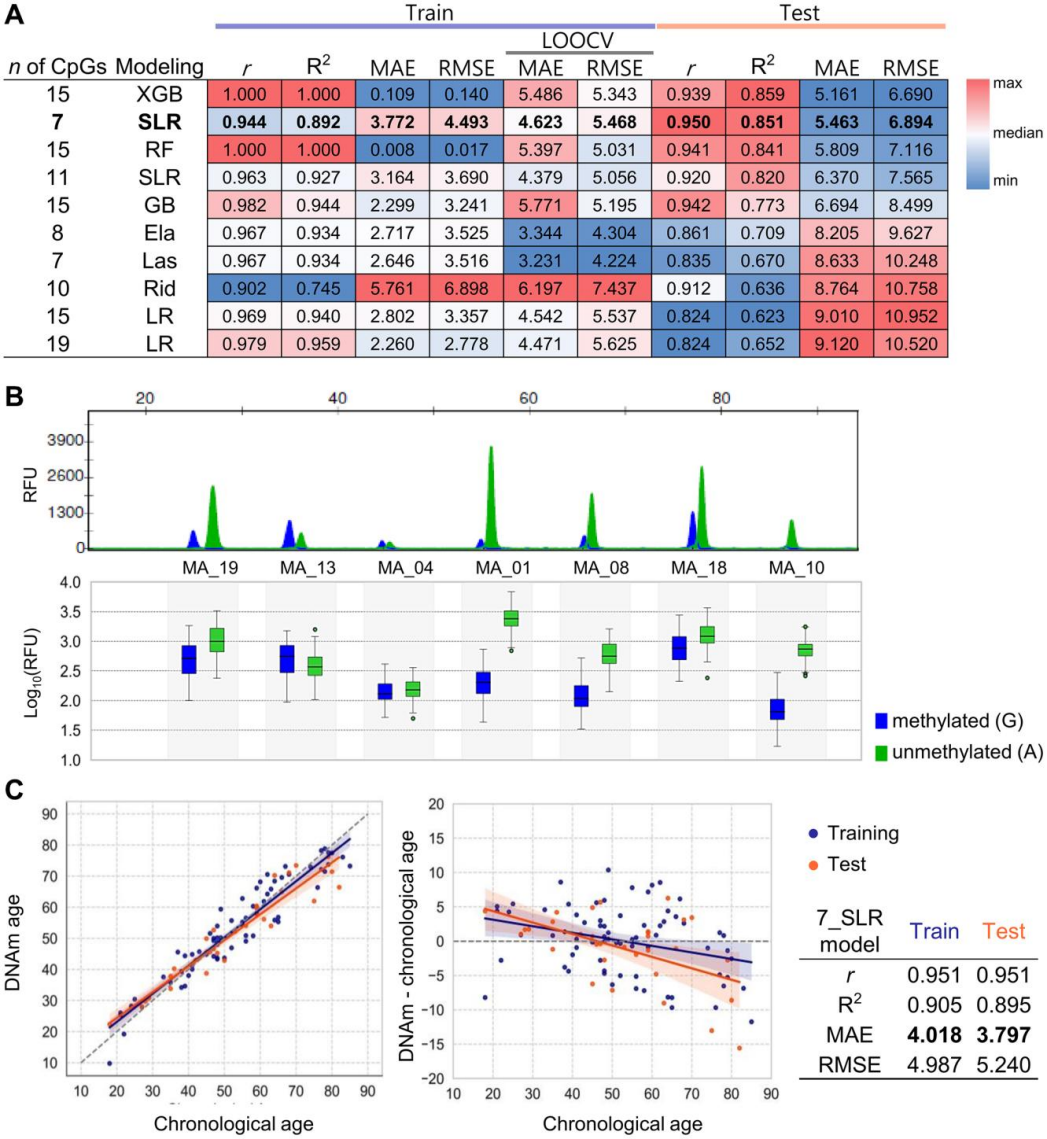
**Development and validation of age prediction models using the SBE system.** (**A**) Performance heatmap of 10 machine learning models based on the SBE platform. Models differ by algorithm (LR, SLR, Ela, Las, Rid, RF, GB, XGB) and number of CpGs used. The performance metrics such as *r*, R^2^, MAE, and RMSE values were calculated for both training and test sets. A total of 68 samples were used, with 43 assigned to the training set and 25 to the test set. An additional 35 samples were later included, resulting in a final dataset of 103 samples, consisting of 71 samples for training and 32 for testing, which were used for the final modeling. The heatmap displays relative values using a red-to-blue gradient for each column criterion. The top-performing SBE model is shown in bold. (**B**) Electropherogram of a unified SBE system using seven CpGs selected from both NGS and SBE models. Capillary electrophoresis was performed using a 3500 genetic analyzer (upper plot). The log_10_(RFU) values for each peak are shown in a box plot, displaying the mean ± SEM (lower plot). Blue fluorescence represents Guanine (G), representing methylated cytosine, while green fluorescence indicates Adenine (A), representing unmethylated cytosine. (**C**) Age prediction performance of the best-performing SBE model. The best model’s accuracy was assessed by comparing predicted age with chronological age for 103 individuals in the training and test sets, represented by orange and blue points, respectively (left plot). Residuals between DNAm age and chronological age were plotted (right plot). Regression lines and 95% confidence intervals are shown. Performance metrics for the model are summarized in the accompanying table.

To identify the most significant markers, 10 distinct models were developed using the remaining 19 CpGs with various machine learning techniques. All models achieved strong performance, with *r* > 0.902 and R² > 0.754 in training set, and *r* > 0.824 and R² > 0.623 in the test set (Figure 4A). LOOCV applied to the training set resulted in MAEs ranging from 3.231 to 6.197 years, while the test set showed MAEs between 5.161 and 9.120 years. Stepwise linear regression (SLR) identified a 7 CpG subset, overlapping with the top-performing 21CpG_Ela model derived from NGS data. The SLR model provided the best balance of predictive accuracy and practical simplicity, achieving an MAE of 5.463 and RMSE of 6.894 in the test set (Figure 4A). Three of these 7 CpGs (MA_13, MA_18, MA_19) coincided with previously identified MEAT markers, while the other four (MA_01, MA_04, MA_08, MA_10) were novel to this study.

To ensure accuracy and usability, a unified SBE system was established, targeting only the 7 CpGs (Figure 4B). Utilizing this consolidated system, methylation data from 103 samples were collected, and split into a training set (70%, *n* = 71) and a test set (30%, *n* = 32). The SLR_7CpG model underwent additional optimization, improving predictive accuracy with *r* values of 0.951 for both sets. The refined model achieved MAEs of 4.018 years in the training set and 3.797 years in the test set, demonstrating strong robustness for age prediction (Figure 4C). The coefficients and intercept for the final model are presented in Table 2.

### Assessment of histological applicability and model validity

**Figure 5:**
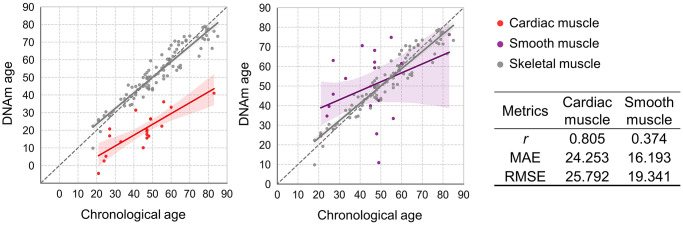
**Applicability of the best-performing SBE model to cardiac and smooth muscle tissues.** Plots compare DNAm age and chronological age using the best SBE model across tissue types. Left: skeletal (gray) vs. cardiac muscle (red, *n* = 19); right: skeletal vs. smooth muscle from uterus (purple, *n* = 19). Skeletal muscle samples (*n* = 103) are shown in gray on both plots. Solid lines represent the regression relationships for each tissue type, with 95% confidence intervals shaded in corresponding colors. Performance metrics for each tissue are summarized in the accompanying table.

To confirm the model’s accuracy and versatility, we expanded the evaluation to include different muscle tissue types. Since tissue-specific cellular composition leads to unique methylation patterns, the model’s performance was tested on cardiac and smooth muscle samples from the heart and uterus, respectively. When the SBE-based skeletal muscle age prediction system (SLR_7CpG) was applied, the heart yielded a high MAE of approximately 24.3 years, indicating poor performance (Figure 5). Similarly, the uterus demonstrated limited accuracy with a MAE of approximately 16.2 years (Figure 5). To assess tissue specificity, EPIC array data were analyzed using linear regression to examine the relationship between chronological age and beta values for the 20 skeletal muscle-specific CpG markers in cardiac and PM muscle tissues. Except for MA_10 and MA_19, the R² values revealed substantial differences between cardiac and skeletal muscle types ranging from 0.261 to 0.683 (Supplementary Figure 8). These findings confirm that the 20 CpG markers and the prediction model are highly specific to skeletal muscle. The diminished accuracy in cardiac and smooth muscle likely arises from differences in cellular composition, such as myocardial cells in the heart and uterine myocytes in the myometrium, which create distinct methylation profiles.

To evaluate the robustness of the assay, we examined the effect of varying DNA input amounts on methylation accuracy and age prediction. Methylation values were calculated from the ratio of methylated to unmethylated sequences generated via PCR amplification. The methylation consistency of the 7 core CpGs included in both SBE and NGS models was assessed using artificially methylated DNA controls (Supplementary Figure 9). The average R2 values were 0.9922 for NGS and 0.9871 for SBE, demonstrating minimal PCR bias. Importantly, when DNA inputs were at least 1.67 ng, the median methylation differences remained under 5% for all markers, except MA_04 in the SBE system (Supplementary Figure 10A). However, MA_13 and MA_18 in the NGS system, as well as MA_19 in the SBE system, showed comparatively greater variability, with standard error of the mean (SEM) values exceeding 0.01 in methylation differences at the 1.67 ng input level. Consistent with previous reports, DNA inputs of at least 10 ng showed reliable performance [28]. Reducing DNA input from 13.33 ng to 6.67 ng increased the MAE by 1.46 years in the SBE model and 2.61 years in the NGS model, both of which were constructed using the same set of 7 CpG markers (Supplementary Figure 10B). Collectively, these results highlight the importance of controlling DNA input to maintain prediction accuracy, with MAEs remaining below 6 years.

## DISCUSSION

Skeletal muscles are composed of various types of fibers, generally classified into six types (I, IC, IIC, IIA, IIAB, and IIB) based on standard myofibrillar adenosine triphosphatase histochemistry [29]. Most skeletal muscles possess a mixed fiber composition, with slow-twitch fibers (Type I) and fast-twitch fibers (Type II) being the most prevalent [12, 30]. Despite this diversity, studies investigating skeletal muscle have primarily concentrated on the VL muscle, which features a heterogeneous distribution of Type I, IIA, and IIB fibers [19]. This heterogeneity has been reported to reflect significant variability in fiber composition, which may contribute to sex-specific differences and complicate the interpretation of fiber-type-specific molecular and epigenetic alterations [29]. These complexities highlight the need to elucidate the molecular and epigenetic characteristics of individual muscle fiber types and specific muscle groups, particularly in research concerning muscle aging and functional regulation. Conversely, the PM muscle in broiler chickens, which is composed almost exclusively of Type IIB fibers, represents a highly homogeneous system, making it suitable for studying the unique aspects of fast-twitch fibers [31]. Despite its potential, the comprehensive epigenetic profile of PM tissue is still insufficiently characterized. Within this framework, utilizing methylation profiling of an aging-gradient group of human PM samples offers an opportunity to enhance molecular investigations and gain a more detailed understanding of fiber-type-specific adaptations and dysfunctions linked to aging and disease.

In various mammalian species, especially in rodents such as rats and mice, aging is associated with distinct fiber type transitions, including a shift from Type IIA to Type I in slow-twitch soleus muscles and from Type IIB to Type IIX in fast-twitch muscles [32–34]. In contrast, in humans, the proportion of fast-twitch MyHC IIa and IIx isoforms diminishes with age, mainly due to the preferential atrophy of Type II fibers, while Type I fibers are relatively maintained [35–38]. Thereby, histochemical myofibrillar type profiles appear to remain relatively stable during aging, despite underlying structural and functional changes [30, 39, 40]. These observations suggest that aging primarily impacts muscle functionality and integrity through the selective atrophy of Type II fibers rather than through pronounced fiber-type switching. Notably, Type IIB fibers, with low oxidative and high glycolytic capacity, are more susceptible to aging-related changes than Type I fibers, which have greater oxidative capacity and resistance to cellular stress [41, 42]. Given these differences in susceptibility, epigenetic changes in Type IIB fibers may further contribute to the age-related decline in muscle function. In this study, we observed that aging in PM muscle samples resulted in 2.4 times more hypermethylated CpGs compared to hypomethylated CpGs, highlighting the potential role of epigenetic modifications in the regulation of muscle function with aging (Figure 1D).

In this study, 20 CpG markers showing age-related hypermethylation or hypomethylation were identified as skeletal muscle-specific markers (Figure 1E, 1F). These markers were associated with 15 genes linked to myopathies and Type IIB fiber function (Figure 2A, 2B). Many of these genes are linked to aging-related functional decline, influencing metabolism, structural integrity, and stress response. Aging process is closely tied to declines in mitochondrial function and oxidative capacity, likely driven by epigenetic changes [43, 44]. Among those genes related to oxidative metabolism, such as *ACTA1*, *IL18BP*, and *HOGA1*, the hypermethylation of *HOGA1* gene may suppress its expression, contributing to the reduced oxidative efficiency observed in Type IIB fibers (Table 1 and Figure 2C). In contrast, glycolytic activity in Type IIB fibers may be sustained to compensate for this decline, as seen in the expression of genes like *CLCN1*, *TPM3*, *TWF2*, and *PIPOX*. Notably, hypomethylation of *TPM3*, which encodes a major component involved in glycolytic muscle contraction, corresponds with increased mRNA expression in the PM muscle samples from elderly individuals (Table 1 and Figure 2C). Additionally, genes such as *CDKN2B* and *KIF15* impact energy metabolism, with hypomethylation of *CDKN2B* potentially promoting muscle cell senescence. Structural genes including *ZNF549*, *RUFY3*, and *SLC44A2* are involved in muscle fiber identity, with the hypermethylation of *SLC44A2* potentially disrupting cellular transport, further contributing to muscle deterioration. To provide a broader aging perspective, we aligned these genes with the hallmarks of aging, as summarized in Supplementary Table 4 [31].

FOXS1 regulates protective stress-response pathway in aging muscles, while SLPI acts as an anti-inflammatory mediator and controls cell proliferation. Despite expectations that transcriptional of these genes would decline with age, we observed increased expression in PM muscle samples from older individuals (Table 1, Figure 2C). This may reflect the influence of the myofibrotic phenotype. Similarly, the pro-IL-18BP exhibited increased mRNA expression despite accelerated methylation, underscoring that DNA methylation and gene expression are not invariably inversely related [45]. This discrepancy may be explained by the low CpG density in its promoter region.

Although some CpGs are located within CpG islands in the transcription region, several exhibited a positive association between methylation and gene expression. For example, despite hypomethylation of *ACTA1*, its mRNA expression decreased. Impaired ACTA1 function may reduce contractile efficiency, resulting in an increased dependence on anaerobic pathways. These observations highlight the multidimensional effects of DNA methylation on gene transcription and the intricate interplay of epigenetic mechanisms. This complexity necessitates further investigation into the structural dynamics of gene regulation in aging skeletal muscle, particularly regarding muscle fiber types and specific genetic loci.

Skeletal muscle is composed of various cell types, including satellite cells, myoblasts, and mature muscle fibers, among others [46, 47]. Although the single-cell analysis, which would precisely characterize methylation patterns specific to each cell subtype, was not performed on PM muscle in this study, we employed stringent procedures during tissue sampling to minimize contamination from non-muscle cells. Blood and visually distinguishable adipose tissue were meticulously removed with precision, and despite these rigorous precautions, some degree of sample heterogeneity could have affected the methylation outcomes. To gain a deeper understanding of methylation alterations at a single-cell resolution, future investigations incorporating single-cell epigenomic approaches are warranted. We additionally applied a novel, skeletal muscle-specific epigenetic predictor to cardiac and smooth muscles to examine inter-muscle epigenetic differences (Figure 5). Our findings revealed significant differences in predictive accuracy, suggesting that skeletal, cardiac, and smooth muscles have distinct DNA methylation profiles. The differences could be driven by their developmental origins, functional demands, regenerative capacities, metabolic requirements, and responses to stress [33, 48–51]. These differences highlight the tissue-specific nature of epigenetic regulation.

While previous studies on muscle diseases have focused on genetic mutations, this study offers methylation patterns during natural aging, identifying potential epigenetic factors contributing to muscle loss and disease. For example, sarcopenia predominantly impacts Type IIB fibers, likely due to epigenetic processes such as hypermethylation-driven suppression of anabolic pathways and hypomethylation of genes promoting inflammation [21, 52–54]. In the Hertfordshire Sarcopenia Study, comparison of the muscle transcriptome between older individuals with sarcopenia and age-matched healthy controls revealed that mitochondrial dysfunction was the predominant transcriptional signature associated with sarcopenia [55]. In this context, mitochondrial dysfunction–related genes such as *SLC44A2*, *TPM3*, *PIPOX*, and *DHDPSL*, which are represented among the 20 CpGs identified in our study, may play a contributory role in the pathophysiology of sarcopenia. Additionally, DNA methylation, histone modifications, and non-coding RNAs may act coordinately to regulate gene expression in models of skeletal muscle atrophy [56]. This raises the possibility that CpG loci mapping to genes involved in inflammation, stress response, and metabolic regulation, including the 20 CpGs identified in our study, could predispose skeletal muscle to accelerated atrophy under age-associated stressors. In this regard, if the methylation status of the 20 age-related CpG markers identified in this study can be precisely modulated, it may help alleviate or delay muscular diseases such as sarcopenia. While technically demanding, modulating targeted DNA methylation will offer a potential therapeutic avenue. This underscores the importance of epigenetic interventions in the prevention or treatment of aging-related muscle diseases, paving the way for future research into targeted epigenetic therapies.

From a practical perspective, developing an epigenetic age predictor based on skeletal muscle holds significant promise, especially in forensic science. Unlike Voisin’s MEAT clock, which is based on biopsy-derived VL muscle samples from European populations, the models introduced in this study mark the use of post-mortem PM muscle samples from a Korean population (Table 2). While the array-based MEAT model showed considerable prediction accuracy in Korean PM muscle samples [20], its broader application is hindered by stringent requirements for DNA quantity, high costs, and limited practical feasibility. By contrast, the newly developed SBE and NGS models are expected to overcome these limitations by optimizing DNA input, the number of CpG markers, and cost-effectiveness. Notably, our models could aid in identifying victims by enabling accurate estimation of DNA methylation age from degraded human remains in forensic science.

In summary, this study identified 20 skeletal muscle-specific CpG markers with aging by analyzing genome-wide methylation data from pectoralis major muscle autopsy samples using the EPIC array. These markers are expected to be pivotal in furthering insight into the genetic and epigenetic underpinnings of aging and their connection to age-related genetic disorders. Based on these markers, skeletal muscle-specific age prediction models with high prediction accuracy were constructed using both SBE and NGS platforms. These models exhibited robust performance, demonstrated skeletal muscle tissue specificity, as evidenced by the reduced predictive accuracy in smooth and cardiac muscle tissues, and showed cost-effectiveness, highlighting their practical utility. The methylation profiles and prediction tools developed in this study present valuable resources for researchers, clinicians, and forensic scientists, offering novel insights for investigating skeletal muscle biology, age-related muscular disease, and human aging.

## MATERIALS AND METHODS

### Study samples

Ethical approval for this study was obtained from the Institutional Review Board of Seoul National University Hospital (IRB NO. C–1912–053-1087). PM muscle samples were collected via autopsy from 103 deceased human individuals of South Korean descent, aged 18 to 85 years, comprising 80 males and 23 females (Supplementary Figure 1). Cardiac (heart) and smooth muscle (uterus) samples were also collected from the 23 female donors. To minimize the effect of post-mortem interval (PMI) on DNA integrity and methylation status, samples were isolated from non-lesioned tissue areas of uncompromised tissues to ensure integrity with PMIs ranging from 1 to 12 days, averaging 2.4 days. After excision, all tissue samples were promptly stored at −80°C to preserve their molecular stability until further processing.

### Genomic DNA extraction

Genomic DNA (gDNA) was extracted from tissue samples using the QIAamp^®^ DNA Mini Kit (Qiagen, Hilden, Germany). Key adjustments to the protocol included processing 25 mg of tissue with a 4-hour lysis step, optimized to maximize extraction efficiency. The purified gDNA was quantified using the Qubit^™^ dsDNA HS Assay Kit on a Qubit^™^ Flex Fluorometer (Thermo Fisher Scientific, Waltham, MA, USA) and subsequently stored at −20°C.

### Identification of skeletal muscle-specific aging-related CpG sites

To identify age-associated CpG sites in skeletal muscle, DNA methylation profiles from 23 male PM muscle samples were analyzed, including twenty from the GSE244996 dataset and three newly generated for this study (GSE294234). Genome-wide DNA methylation profiling was conducted using the Illumina Infinium Methylation EPIC BeadChip array version 1 (Illumina, San Diego, CA, USA) in collaboration with Macrogen Inc. Bisulfite conversion was performed on 250 ng of gDNA to facilitate methylation-specific analysis, followed by amplification to ensure sufficient DNA quantity for hybridization. The DNA was enzymatically fragmented, hybridized to sequence-specific probes on the array, and underwent single-base extension incorporating labeled nucleotides. After staining, arrays were scanned on an Illumina scanner. Fluorescent intensity data were extracted and preprocessed in R with the lumi package to perform background correction and dye bias equalization. Probes with detection *p*-value ≥ 0.05 in more than 25% of samples or missing values (NA) in any sample were excluded. Beta Mixture Quantile (BMIQ) normalization was implemented to standardize methylation values. The resulting Beta and M-values were calculated, and CpG site selection was further refined using statistical criteria, including detection *p*-value, a maximum beta value difference (Δβ) and R^2^ values from regression between beta values and chronological age. For comparison, VL muscle data from GSE114763 (*n* = 8) and GSE50498 (*n* = 47), and cardiac muscle data from GSE244996 (*n* = 20), were obtained from the Gene Expression Omnibus (GEO).

### Genomic analysis of aging-associated genes

To identify enriched signaling and metabolic pathways, as well as to predict the activation or inhibition of upstream regulators and evaluate downstream consequences on diseases, biological functions, and phenotypes, the IPA plugin version 23.0 (Qiagen, Frederick, MD, USA) was utilized. Inferences for expression and pathway analysis were made indirectly using raw *p*-values and regression coefficients obtained from EPIC array data. Statistical significance was determined by a −log_10_ (*p*-value) > 1.3, corresponding to a *p*-value < 0.05.

### mRNA expression analysis by quantitative real-time PCR

Total mRNA was extracted from 10 mg of muscle tissue using QIAzol and the RNeasy Plus Universal Mini Kit (Qiagen) and quantified using a NanoDrop spectrophotometer (Thermo Fisher Scientific, Waltham, MA, USA). Relative mRNA expression levels were analyzed through Real-Time quantitative PCR (RT-qPCR) on a CFX Connect Real-Time PCR System (Bio-Rad Laboratories, Hercules, CA, USA), utilizing the SensiFAST SYBR Lo-ROX One-Step Kit (Meridian Bioscience, Cincinnati, OH, USA) and custom-designed primer sets (Supplementary Table 5). To better assess the correlation between methylation of the target CpGs and mRNA transcription levels, custom primers were designed to amplify mRNA regions encompassing or adjacent to the 20 CpG sites of interest.

### Bisulfite conversion

Bisulfite conversion was performed on 200 ng of gDNA using the EZ DNA Methylation-Lightning Kit (Zymo Research, Irvine, CA, USA), following the manufacturer’s instructions. The resulting bisulfite-converted DNA (bcDNA) was eluted in 15 μl of buffer, yielding 13.33 ng/μl, assuming 100% recovery [57]. Sensitivity was assessed by serial dilutions down to 12.5 ng of gDNA input, resulting in 0.83 ng/μl of bcDNA. To validate methylation accuracy, standards were created by mixing fully methylated (100%) and unmethylated (0%) DNA in 10% increments using the EpiTect PCR Control DNA Set (Qiagen).

### Primer design for methylation analysis

Bisulfite sequencing primers were designed using Pyromark Assay Design Software version 2.0 (Qiagen) and Bisearch (http://bisearch.enzim.hu), with optimal parameters including a melting temperature range of 56–62°C, 100 bp amplicon size, 25 bp primer length, and exclusion of degeneracy and secondary structures (Supplementary Table 6). SBE primers with extended T-tails for multiplex analysis were designed (Supplementary Table 7), and the final primer sets used in the model are detailed in Supplementary Table 8.

### Bisulfite sequencing using single base extension

For the SBE assay, 1 μL of bcDNA (13.33 ng/μL) was amplified in a 20 μL multiplex PCR reaction containing 2 μL of 10× Gold ST*R Buffer (Promega, Madison, WI, USA), 3.5 U of AmpliTaq Gold DNA Polymerase (Applied Biosystems, Foster City, CA, USA), and sequence-specific primers. The PCR protocol began with an initial denaturation at 95°C for 11 minutes, followed by 34 cycles of 94°C for 20 s, 56°C for 60 s, and 72°C for 30 s, concluding with a final extension at 72°C for 7 min. The amplified PCR products were first purified using EXOSAP-IT^™^ (Applied Biosystems) at 37°C for 45 min, then heat-inactivated at 65°C for 15 min. DNA methylation detection by SBE was performed in a 10 ul reaction mixture containing 1 μL of 10× SNaPshot Multiplex Ready Reaction Mix, 2 μL of 5× BigDye^™^ Terminator v.1.1 and 3.1 Sequencing Buffer (both from Applied Biosystems), SBE primers, and 1 μL of purified PCR product. SBE thermal cycling conditions consisted of 96°C for 10 s, 50°C for 5 s, and 60°C for 30 s, repeated for a total of 28 cycles. The SBE products were then purified using recombinant SAP (Applied Biosystems) under the identical conditions as the previous purification. All PCR and SBE reactions were conducted on a Veriti 96-Well Thermal Cycler (Applied Biosystems, Waltham, MA, USA), and sequencing was performed using a 3500 Genetic Analyzer (Thermo Fisher Scientific) equipped with a 36 cm capillary and POP-4 polymer.

### Bisulfite sequencing using next generation sequencing

For targeted NGS, libraries were prepared using the KAPA HyperPrep Kit, KAPA Universal Adapters, and KAPA Unique-Dual Indexed (UDI) Primer Mixes, in accordance with the manufacturer’s guidelines provided in the KAPA HyperPrep Kit technical datasheet. Initially, 13.33 ng of bcDNA underwent targeted multiplex PCR with specific primer sets (Supplementary Table 3). PCR products exceeding 300 ng were then subjected to end-repair and A-tailing procedures. The reaction mixture was incubated with a universal adapter at 20°C for 1 hour. Following adapter ligation, each library was purified using AMPure XP beads (Beckman Coulter, Brea, CA, USA). Libraries were then amplified using UDI primer mixes through 13 cycles of PCR and subsequently purified again with AMPure XP beads. The concentration and quality of the libraries were assessed using the 2100 Bioanalyzer and 4150 TapeStation (Agilent, Santa Clara, CA, USA), and all samples were pooled into a single batch for further processing. Double-size selection was performed using SPRIselect beads (Beckman Coulter) to ensure the desired fragment size. The final library was quantified using KAPA Library Quantification Kits (Roche, Basel, Switzerland), following the provided instructions. The pooled library was adjusted to 4 nM, denatured with 0.2 N NaOH, and adjusted to 20 pM with Illumina prechilled hybridization buffer. The denatured library was subsequently diluted to 7–9 pM with a spike-in of 2.5% PhiX control v.3. Sequencing was performed on an Illumina MiSeq system using the MiSeq Reagent Kit v.3, with a 2 × 300 cycle configuration (Verogen, San Diego, CA, USA).

### Methylation data processing

SBE methylation data were analyzed using GeneMapper Software 5 (Thermo Fisher Scientific). Methylation values were calculated from the peak height, representing the relative fluorescence units (RFU) of C or G nucleotides corresponding to methylated DNA, normalized by the total DNA, determined as the sum of C+T or G+A nucleotides. Raw sequencing data from NGS (FASTQ files) were processed on CLC Genomics Workbench 21.0.5 (Qiagen). The analysis workflow included merging paired-end reads, trimming low-quality bases based on Phred scores reflecting a 1% error probability, and excluding reads containing two or more ambiguous bases or measuring less than 10 bp in length. Processed reads were aligned to the reference genome (Human GRCh38), and methylation calling was performed, omitting loci with coverage below 20. The resulting methylation values were utilized for subsequent machine learning modeling.

### Machine learning

Machine learning analyses to develop age prediction models were performed using Python 3.8.8. Linear regression (LR), stepwise linear regression (SLR), Lasso Regression (Las), ridge regression (Rid), and elastic-net regression (Ela) were implemented. Tree-based decision models, including XGBoost (XGB), gradient boosting (GB), and random forest (RF), were implemented using the Scikit-learn and XGBoost libraries in Python. Hyperparameters were optimized with GridSearchCV, and model performance was evaluated with metrics including the Pearson correlation coefficient (*r*), R-squared (R²), mean absolute error (MAE) and root mean square error (RMSE).

### Statistical analysis

Statistical comparisons for mRNA expression were performed using the Chi-square test and the unpaired *t*-test. Data are presented as mean ± standard error of the mean (SEM) and were analyzed using GraphPad Prism 5.01 (GraphPad, La Jolla, CA, USA). Levels of statistical significance are denoted by asterisks with the following thresholds: ^*^p ≤ 0.05; ^**^p ≤ 0.01; ^***^p ≤ 0.001.

### Availability of data

All data supporting the findings of this study are available within the paper and its Supplementary files. Raw data underlying the main and supplementary figures and tables have been deposited in the Gene Expression Omnibus (GEO) under accession numbers GSE244996 and GSE294234. A source data file containing statistical analyses and *p*-values is provided as Supplementary Table 1.

## Supplementary Materials

Supplementary Figures

Supplementary Table 1

Supplementary Table 2

Supplementary Tables 3-8

## Abbreviations

NGS: Next-Generation Sequencing
SBE: Single Base Extension
VL: Vastus Lateralis
PM: Pectoralis Major
TSS: Transcription Start Site
UTR: Un-Translated Region
IPA: Ingenuity Pathway Analysis
MAE: Mean Absolute Error
LOOCV: Leave-One-Out Cross-Validation
gDNA: Genomic DNA
BMIQ: Beta Mixture Quantile
GEO: Gene Expression Omnibus
DNAm: DNA methylation
RT-qPCR: Real-Time quantitative PCR
bcDNA: bisulfite-converted DNA
LR: Linear Regression
SLR: Stepwise Linear Regression
Las: Lasso regression
Rid: Ridge regression
Ela: Elastic-net regression
XGB: XGBoost
GB: Gradient Boosting
RF: Random Forest
r: Pearson correlation coefficient
R²: R-squared
MAE: Mean Absolute Error
RMSE: Root Mean Square Error
SEM: Standard Error of the Mean
