# Supplementary material for: Epigenetic aging signatures and age prediction in human skeletal muscle
Source: Aging (Albany NY). 2025 Nov 26;17(11):2809–43. doi: 10.18632/aging.206341 (PMC12705185; doi:10.18632/aging.206341)
Supplement: Supplementary Figures [file aging-17-11-206341-s001.pdf]

SUPPLEMENTARY FIGURES

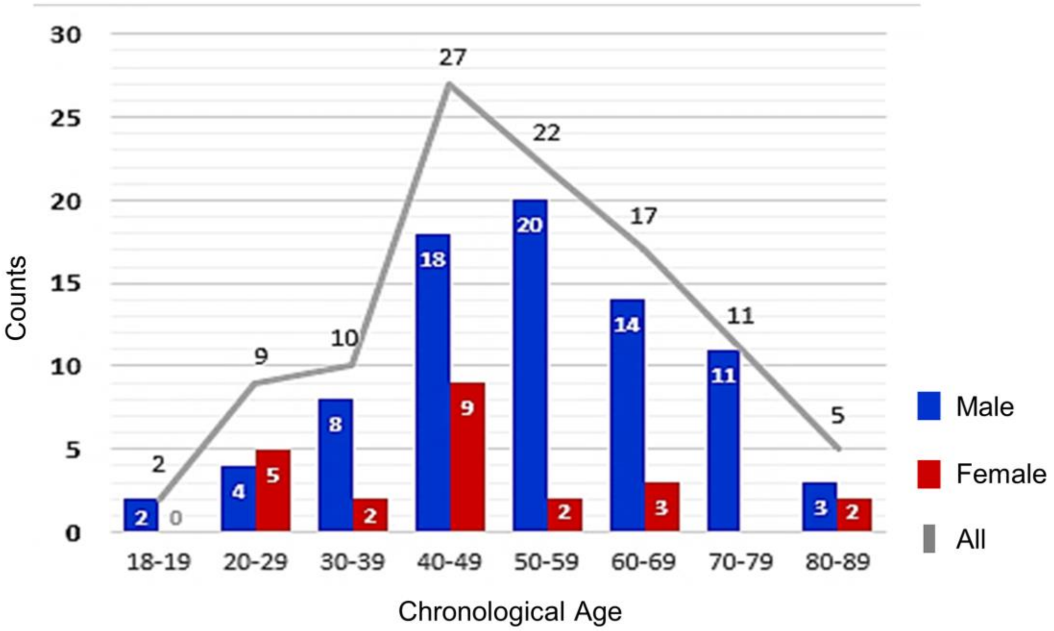

Supplementary Figure 1. Distribution of samples by age group and gender. The distribution of 103 samples across age groups and gender is shown. The age groups and sample sizes are as follows: 18–19 years (n = 2), 20–29 years (n = 9), 30–39 years (n = 10), 40–49 years (n = 27), 50–59 years (n = 22), 60–69 years (n = 17), 70–79 years (n = 11), and 80–89 years (n = 5). Male samples are depicted by blue boxes, and female samples by red boxes. The number of samples in each age group is indicated as the gray lines.

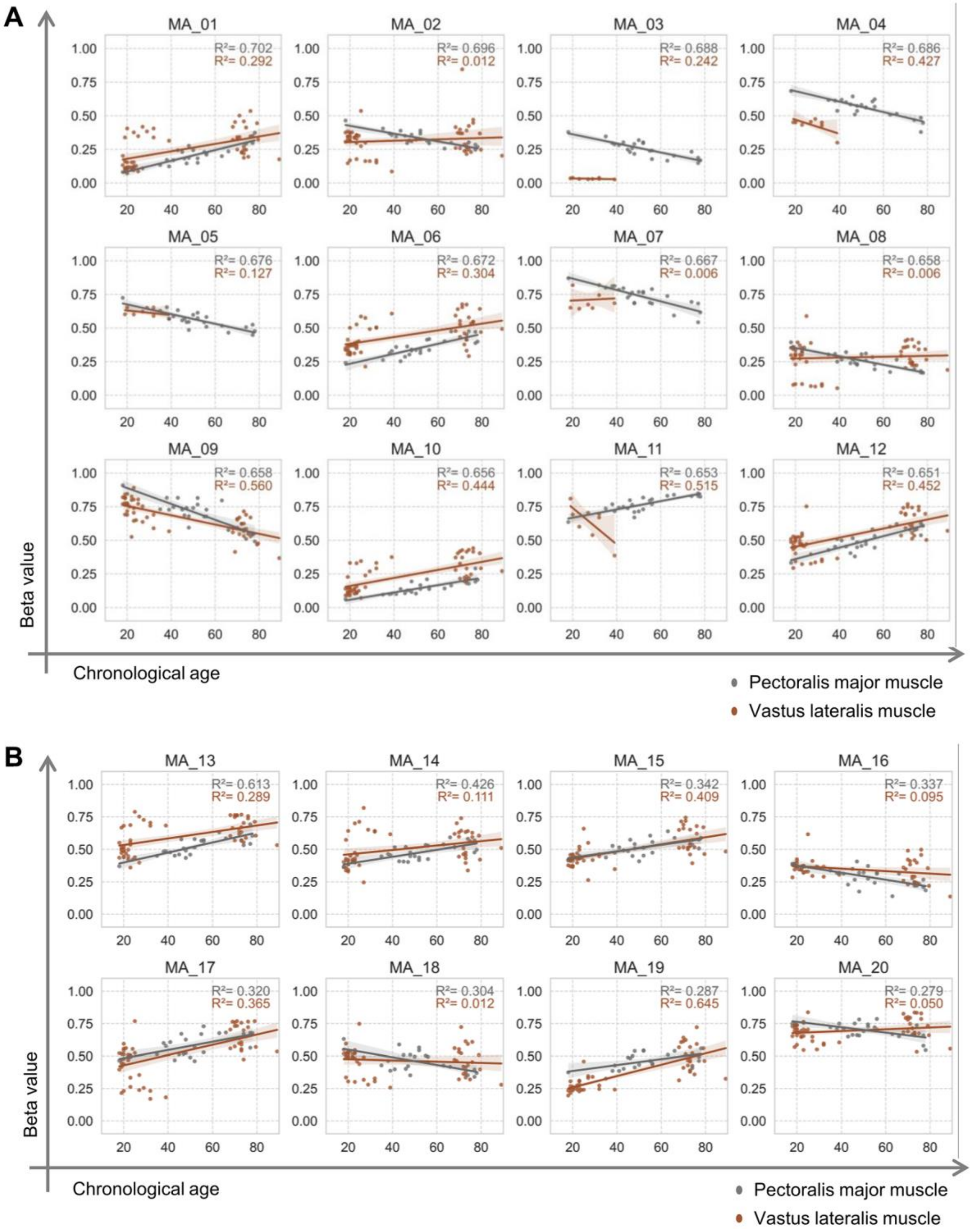

Supplementary Figure 2. Comparison of array beta values between pectoralis major muscle and vastus lateralis muscle across skeletal muscle-specific 20 CpG markers. Linear regression was performed to assess the relationship between beta values (ranging from 0 to 1) and chronological age for 12 newly identified CpG markers (**A**) and 8 CpG markers overlapping with MEAT [11] (**B**). Publicly available datasets were used to assess age-related associations, with linear regression between beta values and age. Vastus lateralis muscle data were obtained from GSE114763 (*n* = 8) using the HM450 array and GSE50498 (*n* = 47) using the HMEPIC array. Both datasets were used for the MEAT, with samples represented as brown dots, and R^2 values highlighted in brown. Pectoralis major muscle data were obtained from GSE244996 (*n* = 20) and GSE294234 (*n* = 3) generated in this study, with samples represented by gray dots and the corresponding R^2 values in gray. Solid lines represent the regression relationships for each tissue type, with 95% confidence intervals shaded in corresponding colors.

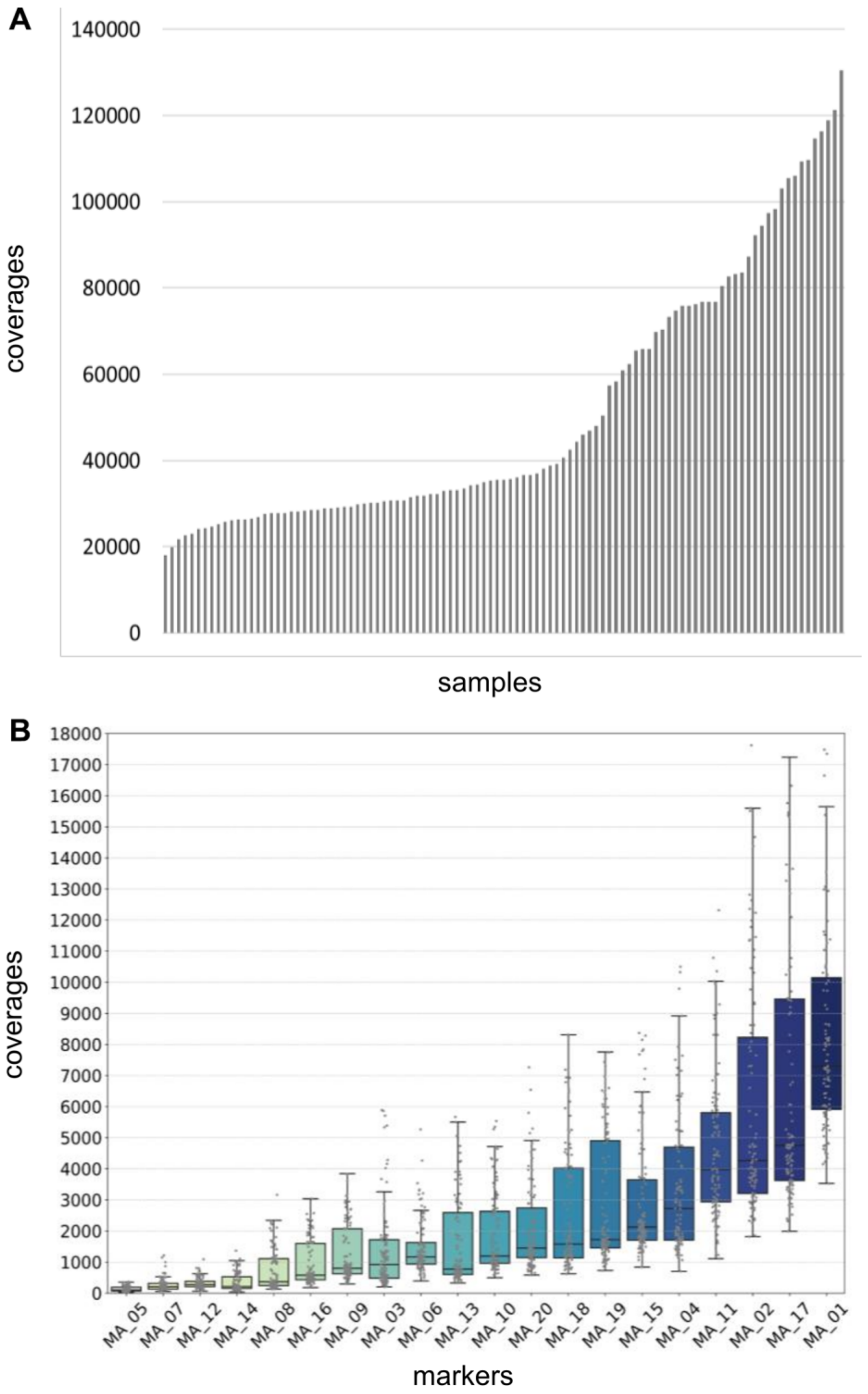

Supplementary Figure 3. Quality assessment of data obtained via NGS. (**A**) Sample Coverage Analysis. Sequencing depth was assessed for all 103 samples, with coverage values plotted on the Y-axis and samples arranged on the X-axis in descending order of coverage. (**B**) Marker Coverage Analysis. Coverage across the 20 target CpG amplicons was evaluated for each marker. Box plots, generated using the Seaborn library in Python, illustrate the distribution of coverage, with gray dots representing individual sample values for each marker.

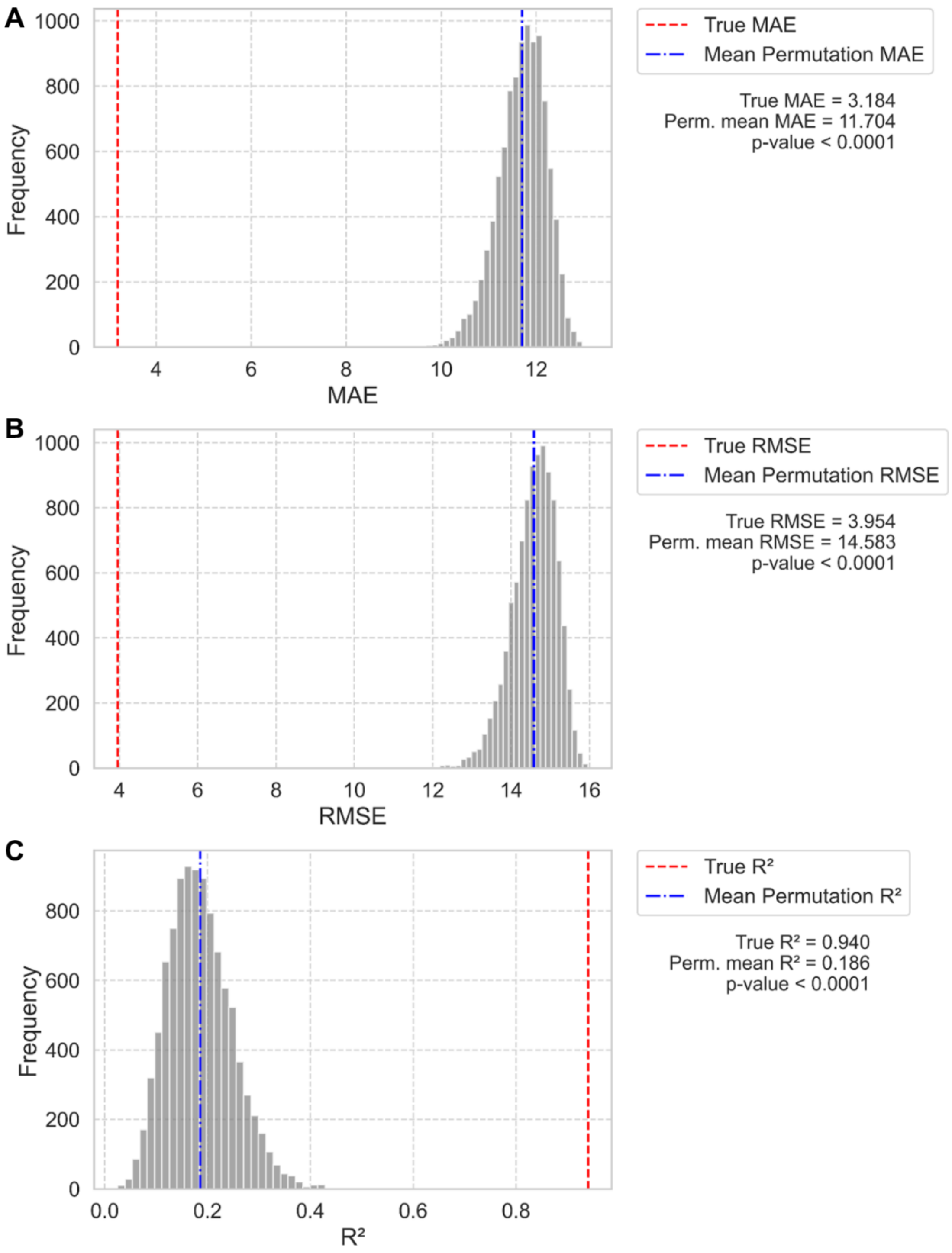

Supplementary Figure 4. Permutation test of the 21_Ela model. Null distributions of prediction accuracies were generated by randomly permuting age labels 10,000 times while keeping the CpG feature matrix fixed. (**A**) Distribution of MAE values under the null. (**B**) Distribution of RMSE values under the null. (**C**) Distribution of R^2 values under the null. The red dashed line indicates the observed performance of the 21_Ela model. The blue distributions represent the null performance obtained from 10,000 permutations.

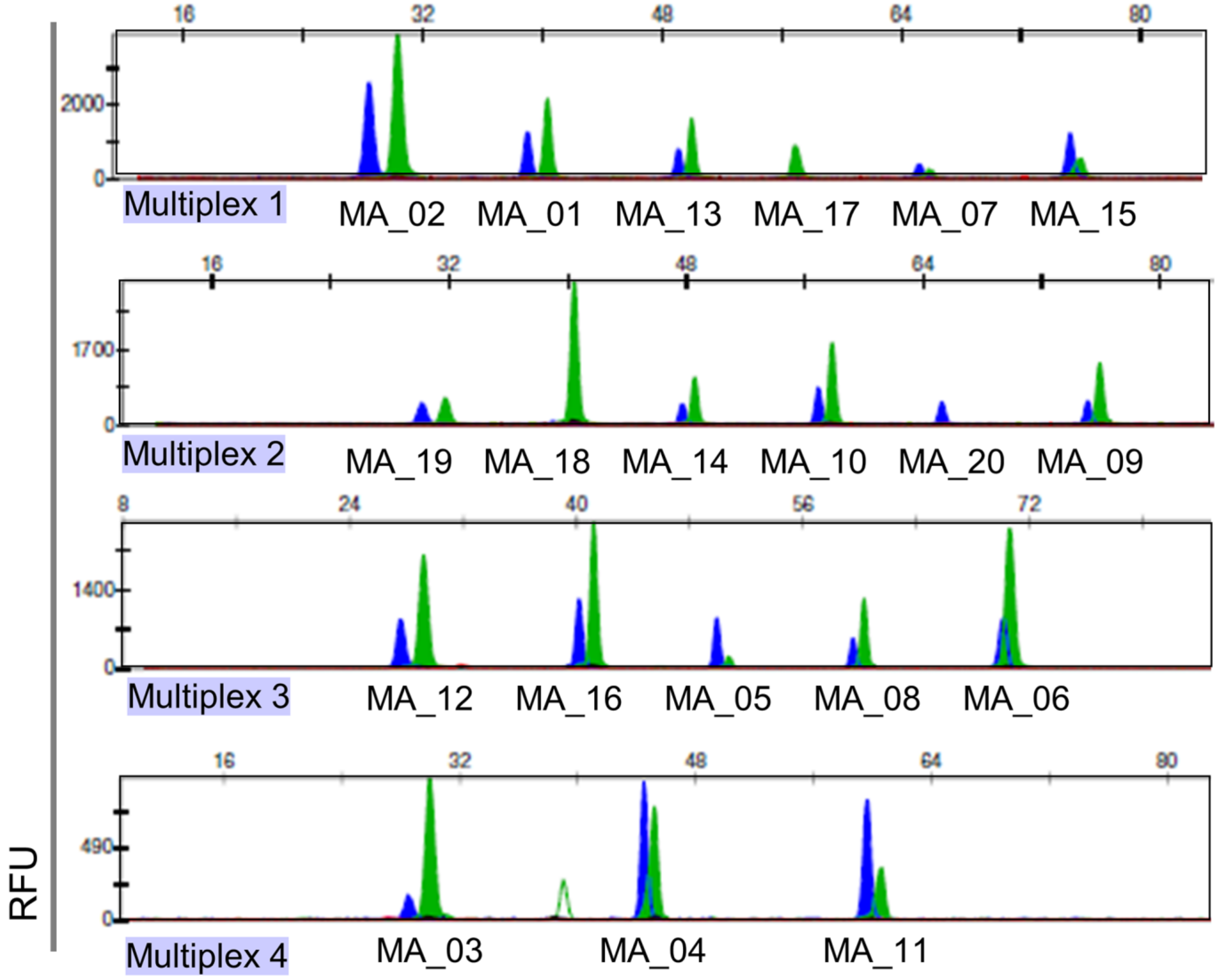

Supplementary Figure 5. Electropherogram of four multiplex groups for muscle-specific aging-related 20 CpGs on SBE. The electropherograms were obtained using a 3500 Genetic Analyzer, showcasing four multiplex groups for the 20 muscle-specific aging-related CpGs. Each group is represented in a separate row. Blue fluorescence corresponds to Guanine (G), indicative of methylated Cytosine (5mC), while green fluorescence represents Adenine (A), signaling unmethylated Cytosine (C). The peaks for each CpG marker are shown, with the x-axis indicating the relative fragment size, and the y-axis representing the relative fluorescence units (RFU). The markers are displayed within each multiplex group, as indicated by their respective labels (MA_01, MA_02, etc.).

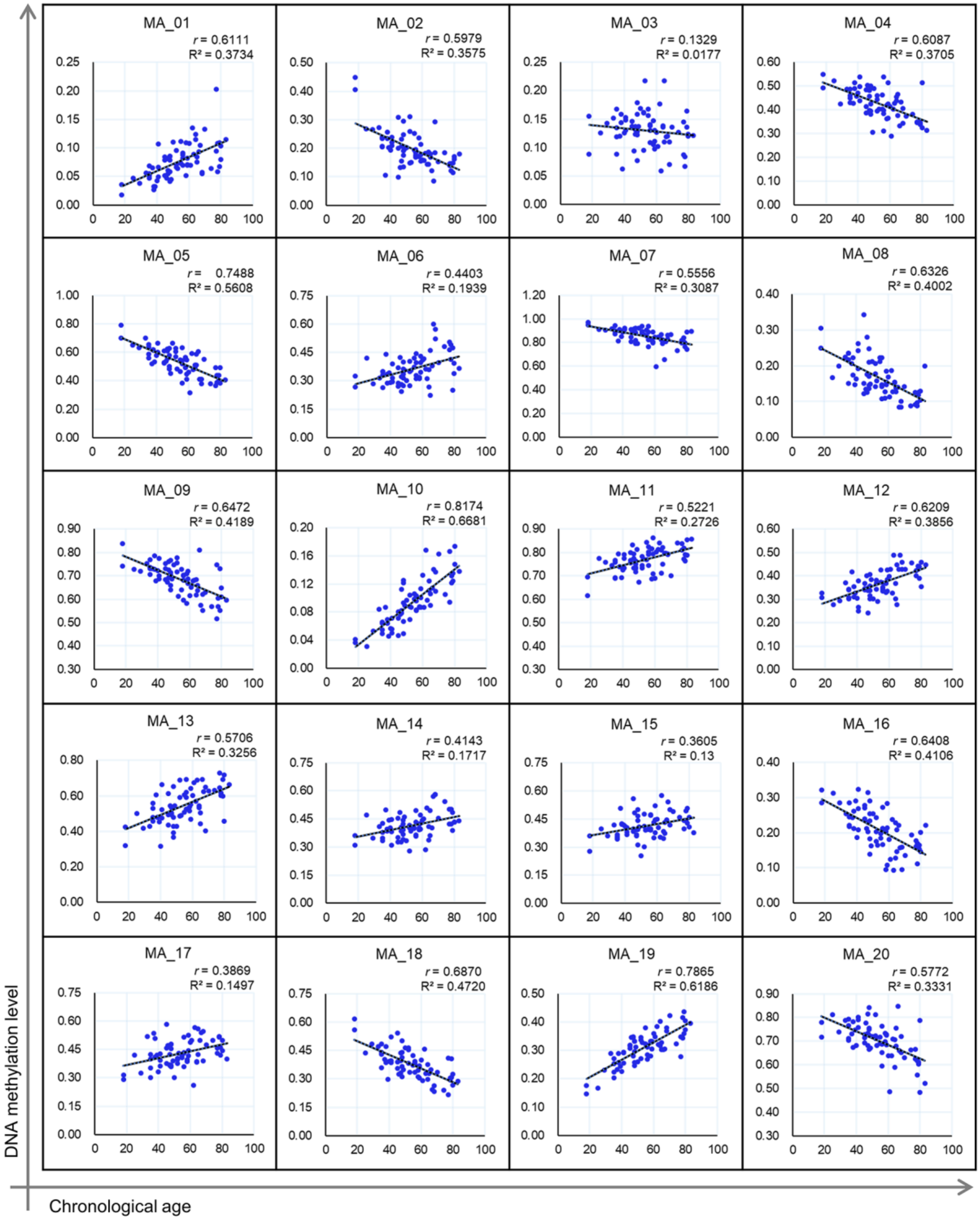

Supplementary Figure 6. Linear regression analysis of 20 muscle-specific age-associated CpG markers. Linear regression was conducted to assess the relationship between DNA methylation levels (ranging from 0 to 1) and chronological age for 20 skeletal muscle-specific CpG markers. Each sample is depicted as a blue dot. The statistical results including correlation coefficient (*r*) and R^2 values for each marker are shown on the respective plots.

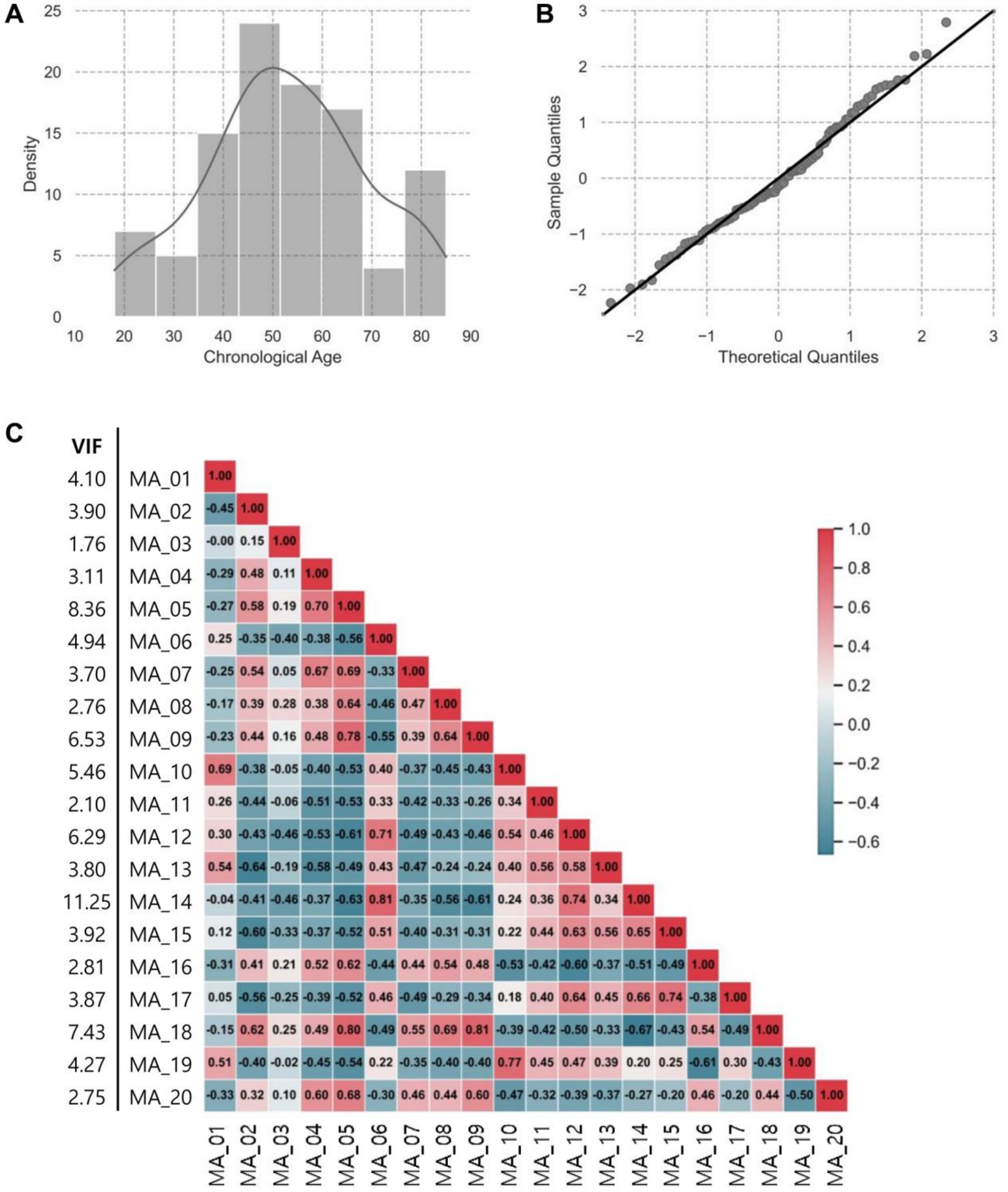

Supplementary Figure 7. Quality assessment of data obtained via SBE. (**A**) Density Estimation and Normal Distribution Check. Density estimation was performed to evaluate the distribution of the data, where the height of each bar represents the density of Y values, not the count. The kernel density estimate (KDE) curve provides a smoothed representation of the data distribution. Skewness (−0.059) and kurtosis (−0.476) values confirmed that the data follows a normal distribution. (**B**) Normality of Residuals in Linear Regression. Linear regression assumes that residuals follow a normal distribution. A Q-Q plot was used to compare the quantiles of the residuals against theoretical quantiles from a normal distribution. Deviations from the diagonal line indicate departures from normality. (**C**) Correlation and Multicollinearity Assessment. A correlation and multicollinearity test were conducted to identify high intercorrelations among independent variables. The heatmap illustrates correlation levels, ranging from high (red) to low (blue). Strong correlations (coefficient ≥0.8) were observed in three variable pairs. Additionally, a variance inflation factor exceeding the threshold of 10 was identified for the MA_14 marker, highlighting potential multicollinearity concerns.

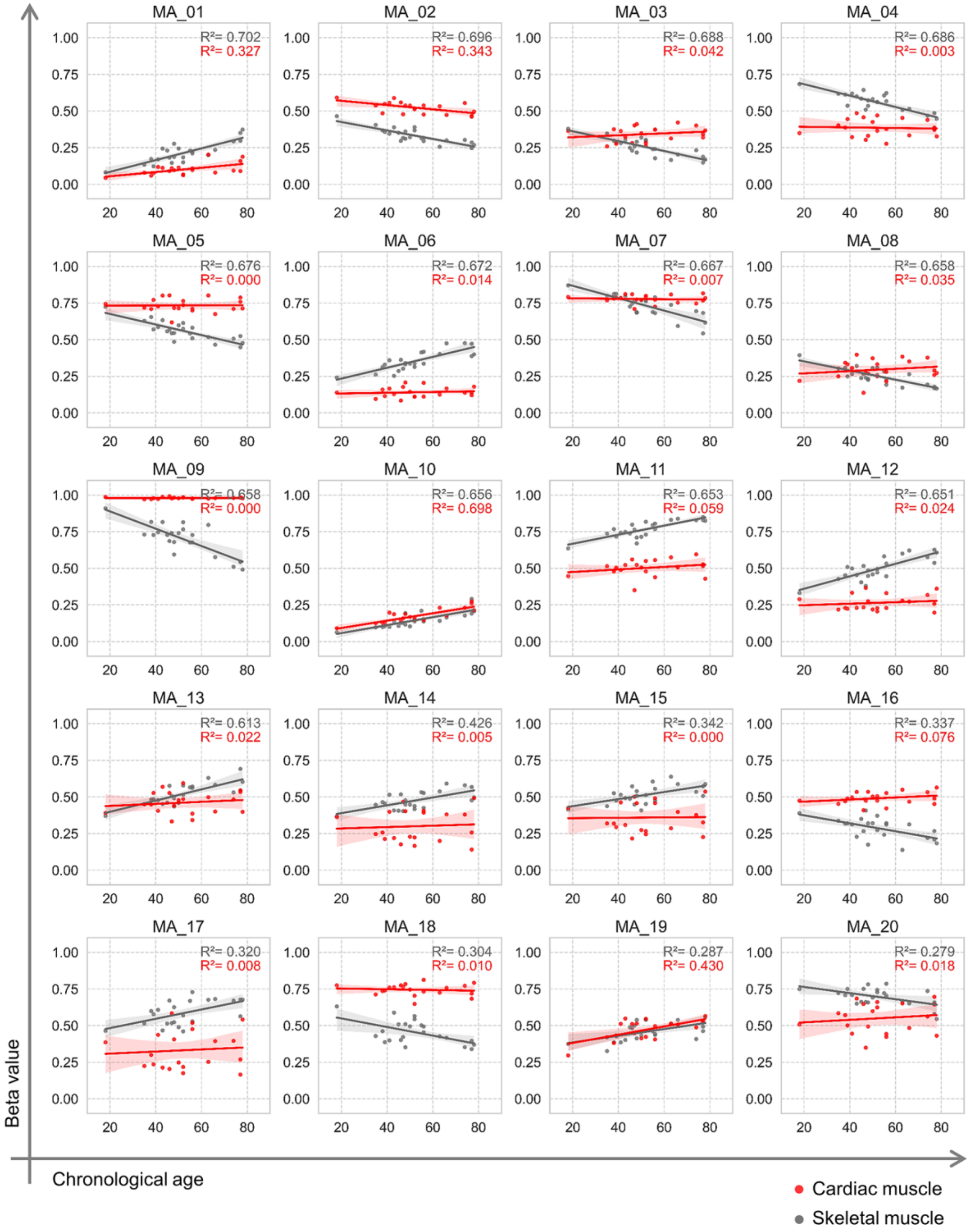

Supplementary Figure 8. Comparison of array beta values between skeletal muscle and cardiac muscle on skeletal muscle-specific 20 CpG markers. Linear regression was performed to assess the relationship between beta values (ranging from 0 to 1) and chronological age for 20 skeletal muscle-specific CpG markers. Pectoralis major muscle data were obtained from GSE244996 (n = 20) and GSE294234 (n = 3) from this study, with samples represented as gray dots and corresponding statistical R^2 values shown in gray. Cardiac muscle data were obtained from GSE244996 (n = 20), with samples presented as red dots, and corresponding R^2 values are shown in red. Solid lines represent the regression relationships for each tissue type, with 95% confidence intervals shaded in their respective colors.

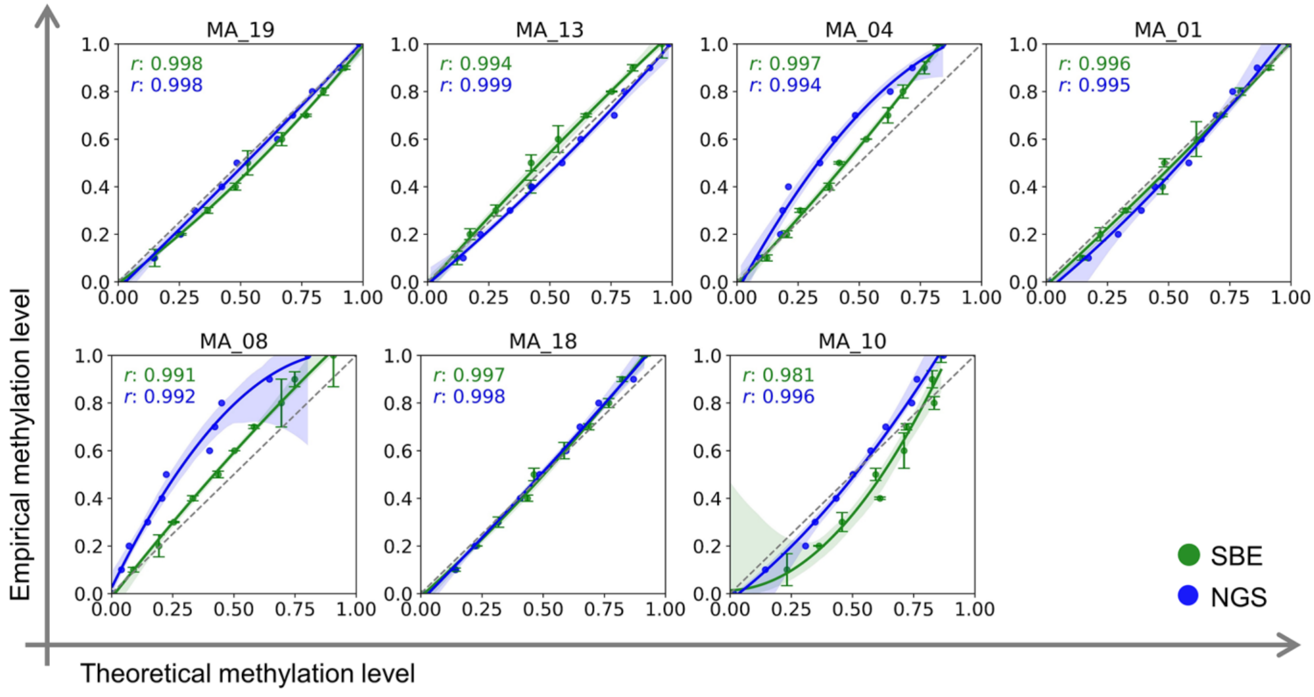

Supplementary Figure 9. Calibration curves for amplification bias in target CpGs. Calibrated curves were generated by analyzing artificially methylated DNA standards (ranging from 0% to 100% in 10% intervals) for target CpGs using SBE (green) and NGS (blue) systems. Polynomial regression lines were fitted to the scatter plots, with the correlation coefficients (*r*) for each marker displayed on the plots. Confidence intervals are displayed as semi-transparent bands around the regression lines. Dots represent the average duplicate observations, with error bars indicating standard deviations.

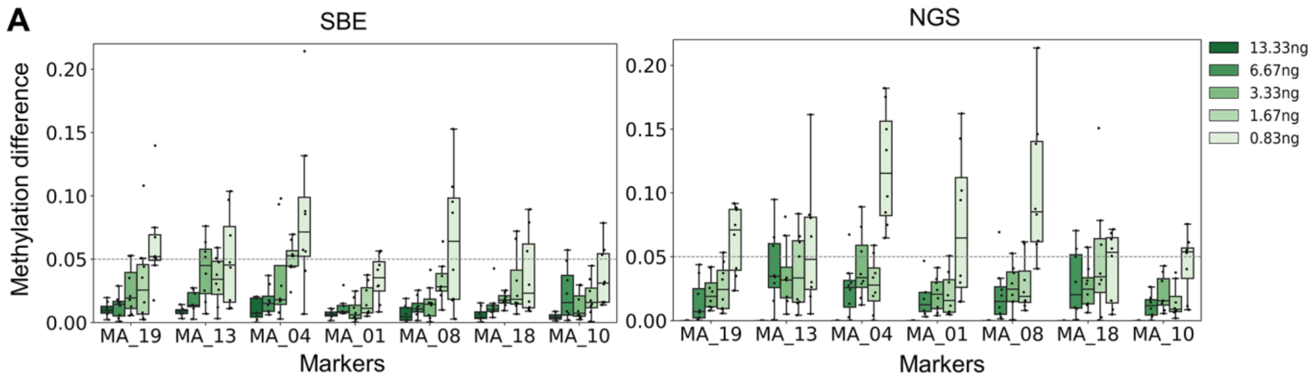

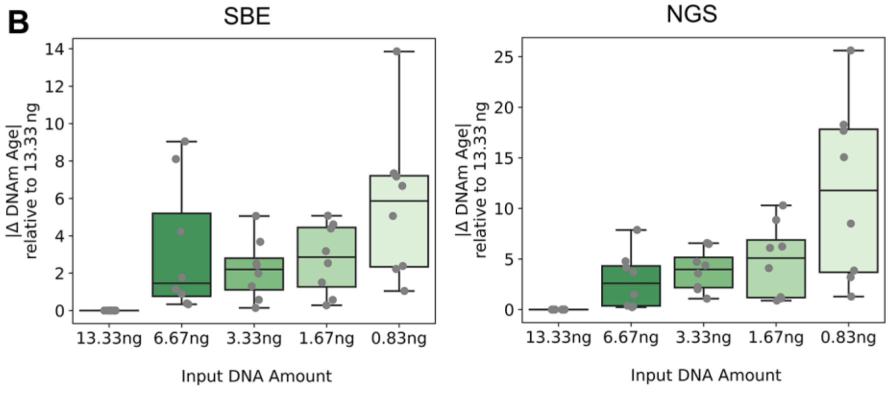

	SBE		NGS	
Input	Median	SEM	Median	SEM
13.33ng	0	0	0	0
6.67ng	1.46	1.246	2.607	0.968
3.33ng	2.2	0.571	3.968	0.721
1.67ng	2.855	0.567	5.095	1.286
0.83ng	5.862	1.448	11.780	3.094

Supplementary Figure 10. Age prediction accuracy across DNA input concentrations using SBE and NGS systems. (**A**) Sensitivity analysis for methylation levels based on DNA input concentration. Sensitivity tests were conducted for the SBE and NGS systems using varying concentrations of bisulfite converted DNA. Methylation levels at 7 core CpGs were compared to a 13.33 ng input, with methylation differences shown on the Y-axis. In the SBE system, a 13.33 ng input was tested in duplicate for all samples, and the mean value was used as the reference. In the NGS system, 13.33 ng input was tested once per sample and used as the reference. Box plots were generated using the Seaborn library in Python, with individual sample values presented as dots. (**B**) Age prediction accuracy based on DNA input concentration. Age prediction was performed using models based on the same set of 7 CpG markers, including the best-performing model in SBE (Figure 4C) and the linear regression model using 7 CpGs in NGS platform (Figure 3B). Box plots, generated with the Seaborn library, illustrate the distribution of age estimation differences across varying DNA input concentrations. The Y-axis represents the difference in age estimates compared to those obtained using the 13.33ng of bcDNA input, while the X-axis denotes the bcDNA input concentrations. The accompanying table summarizes the statistical results, including the median and standard error of the mean (SEM) for each DNA input concentration.
