# Supplementary material for: Epigenetic aging signatures and age prediction in human skeletal muscle
Source: Aging (Albany NY). 2025 Nov 26;17(11):2809–43. doi: 10.18632/aging.206341 (PMC12705185; doi:10.18632/aging.206341)
Supplement: Supplementary Table 2 [file aging-17-11-206341-s003.pdf]

Supplementary Table 2. Detailed formulas of of NGS-based age prediction models.

n of target CpGs	7		16		10		9		9		11	
n of cryptic CpGs	0		5		26		9		12		18	
Modeling	LR		SLR		SLR		Las		Ela		Rid	
	intercept	42.6414	intercept	147.2449	intercept	150.4725	intercept	138.8775	intercept	105.9722	intercept	97.8797
	**chr1:154179209**	-14.7133	**chr1:154179209**	11.0432	**chr1:154179209**	6.3531	chr3:12815282	-13.0311	**chr9:22005565**	17.2336	**chr7:143316521**	-11.4651
	**chr1:229433354**	-3.6950	**chr1:229433354**	-37.8105	**chr1:229433354**	-18.9269	**chr7:143316521**	-23.3704	**chr19:10636467**	-24.7511	chr19:57527208	13.2576
	**chr3:44761961**	152.2639	**chr3:44761961**	124.1519	**chr3:44761961**	79.6482	chr3:44761965	37.1806	**chr19:57527206**	30.6368	chr3:12815285	-11.0047
	**chr7:143316521**	-67.1495	**chr7:143316521**	-6.6488	**chr7:143316521**	12.949	chr11:71998842	-9.5435	**chr1:154179209**	-3.3927	chr3:12815338	-9.7142
	**chr9:22005565**	14.7792404,	**chr9:22005565**	51.7895	**chr9:22005565**	62.1815	**chr2:15920273**	21.3081	chr1:1937587	10.4319	chr3:12815282	-11.8601
	**chr17:29042762**	42.5096	**chr17:29042762**	20.6215	**chr17:29042762**	16.4622	**chr19:10636467**	-18.6653	**chr2:15920273**	14.5369	**chr20:45255106**	-10.8072
	**chr19:57527206**	80.2852	**chr19:57527206**	88.6459	**chr19:57527206**	77.6669	chr1:229433453	38.5060	chr19:57527211	15.3597	chr9:22005577	9.9550
			**chr1:1937681**	-9.5313	chr3:12815426	-97.3917	**chr9:22005565**	21.6437	**chr17:29042762**	38.6166	chr3:12815320	-6.5007
			**chr2:15920273**	30.3163	chr3:12815466	-91.1509	**chr1:229433354**	-1.3860	chr19:57527218	24.8859	**chr17:29042762**	16.4396
			**chr3:12815297**	-7.1624	chr9:22005570	-42.6121	chr9:22005570	-13.4409	**chr7:143316521**	-23.7932	chr3:12815466	-5.6819
			**chr4:70722006**	16.7646	chr1:229433453	103.271	chr3:12815426	-79.0184	chr3:12815282	-29.4345	chr3:12815348	-5.7061
			**chr7:157014781**	-20.6586	chr2:15920278	3.9341	**chr17:29042762**	34.9134	chr1:229433453	12.7462	chr3:44761965	9.5343
			**chr10:97585212**	13.6126	chr9:22005510	-25.3127	chr7:157014845	5.9814	**chr3:44761961**	53.4929	chr19:57527218	15.3461
			**chr11:71998871**	-18.5413	chr19:57527266	838.7422	**chr1:154179209**	0.0000	chr19:57527208	8.2441	**chr9:22005565**	10.5171
			**chr19:10636467**	-35.8907	chr19:10636490	-124.5958	**chr19:57527206**	83.3785	chr3:12815466	-30.3562	chr9:22005510	8.0299
			**chr20:45255106**	1.8774	chr11:71998842	-3.6824	chr3:12815466	-63.1110	chr19:10636497	7.3709	**chr1:229433354**	-4.6144
			chr3:12815426	-102.7828	chr7:157014763	2.5048	**chr3:44761961**	81.6076	**chr1:229433354**	0.0001	chr3:12815426	-7.8846
			chr3:12815466	-72.9541	chr19:10636497	107.2891	chr1:1937587	7.1786	chr3:44761965	29.5532	**chr3:44761961**	13.6991
			chr1:229433453	78.1356	chr7:157014845	47.4632			chr3:12815338	-11.4447	**chr3:12815297**	-10.1598
			chr9:22005570	-67.5298	chr19:10636475	-49.4553			chr3:12815426	-40.0636	chr3:44761956	9.3837
			chr19:57527266	661.9138	**chr7:157014781**	-44.6131			chr3:44761956	9.3494	chr19:10636475	-6.2769
					chr4:70722010	37.4356					chr19:57527211	14.9374
					chr1:229433352	-21.4639					**chr19:10636467**	-11.9319
					**chr20:45255106**	-5.4741					**chr2:15920273**	7.3949
					chr1:1937587	21.7114					chr10:97585099	-7.3458
					chr1:154179292	14.6886					**chr1:154179209**	-7.1598
					chr11:71998932	-24.8091					chr3:12815364	-5.2131
					chr1:1937577	-15.2486					**chr19:57527206**	14.7116
					chr1:1937530	-0.47					chr3:12815299	-8.4735
					chr3:12815299	96.5078						
					chr3:12815282	-69.6433						
					chr3:44761965	94.4413						
					chr3:12815364	-24.7715						
					chr20:45255065	27.9606						
					**chr10:97585212**	10.7563						
					chr3:12815404	-27.7485						

This table summarizes the regression coefficients and intercepts for linear models (Linear regression, Stepwise Linear Regression, Lasso, Elastic Net, Ridge) and the feature importance scores for machine learning models (Gradient Boosting, Random Forest, XGBoost). Each CpG site is indicated by its genomic coordinate including chromosome and position. The number of CpGs (*n*) used in each model is specified at the top of each column. The 20 CpG markers identified in this study are highlighted in bold.

(*Continued*)

n of target CpGs	11		9		15	
n of cryptic CpGs	19		21		49	
Modeling	GB		RF		XGB	
	feature	importance	feature	importance	feature	importance
	chr3:44761965	0.2691	**chr3:44761961**	0.1645	**chr3:12815297**	0.1917
	**chr3:44761961**	0.188	chr3:44761965	0.1045	**chr1:154179209**	0.1077
	**chr19:10636467**	0.1372	chr19:57527218	0.0869	chr19:57527211	0.0996
	chr19:57527218	0.0987	**chr3:12815297**	0.0843	**chr19:57527206**	0.0629
	chr3:12815285	0.0794	**chr19:10636467**	0.0809	chr3:44761956	0.0629
	chr19:57527221	0.0458	chr3:12815285	0.0639	**chr19:10636467**	0.057
	chr3:12815282	0.0232	chr3:44761956	0.0552	**chr3:44761961**	0.0531
	chr19:57527208	0.0217	chr3:12815282	0.0542	chr3:44761965	0.0459
	chr1:229433474	0.0139	**chr19:57527206**	0.047	chr3:12815282	0.0437
	**chr17:29042762**	0.0119	chr3:12815348	0.0299	chr19:57527208	0.0435
	**chr3:12815297**	0.0119	chr19:57527221	0.0276	chr19:10636475	0.033
	**chr19:57527206**	0.0105	chr19:57527208	0.0252	chr1:229433352	0.0289
	**chr9:22005565**	0.0095	chr3:12815338	0.0184	**chr7:143316521**	0.0185
	**chr2:15920273**	0.0085	chr19:57527211	0.0165	chr19:57527218	0.0176
	chr3:12815338	0.008	chr3:12815299	0.0148	chr20:45255065	0.017
	chr3:12815466	0.0077	**chr2:15920273**	0.0143	chr19:57527221	0.0125
	**chr20:45255106**	0.0068	chr3:12815364	0.0088	**chr17:29042762**	0.0085
	chr19:57527266	0.0063	**chr17:29042762**	0.0086	chr2:15920284	0.0076
	chr19:10636475	0.0044	chr1:229433474	0.0055	chr3:12815426	0.0074
	chr3:44762007	0.0044	chr19:57527266	0.0052	chr3:12815348	0.0065
	chr9:22005510	0.0038	**chr9:22005565**	0.0052	chr3:12815285	0.0057
	chr1:154179292	0.0034	**chr1:154179209**	0.0039	chr2:15920278	0.0056
	chr19:57527254	0.0026	chr19:57527254	0.0036	chr3:12815308	0.0052
	**chr11:71998871**	0.0021	chr9:22005570	0.0035	chr1:1937530	0.0052
	chr7:157014845	0.0019	**chr7:143316521**	0.0034	chr9:22005577	0.0048
	**chr4:70722006**	0.0019	chr3:12815426	0.0032	chr3:12815338	0.0048
	chr3:12815364	0.0017	chr3:12815308	0.0032	chr1:229433474	0.0046
	chr4:70722010	0.0013	chr9:22005577	0.0027	**chr9:22005565**	0.0046
	chr3:12815426	0.0013	chr20:45255065	0.0026	chr19:57527266	0.0036
	**chr7:143316521**	0.0013	chr1:1937724	0.0024	chr10:97585099	0.0036
					chr1:154179292	0.0027
					chr19:57527254	0.0024
					chr11:71998842	0.0021
					**chr20:45255106**	0.002
					chr3:12815466	0.0019
					**chr7:157014781**	0.0015
					chr9:22005570	0.0013
					**chr10:97585212**	0.0012
					**chr2:15920273**	0.0011
					**chr1:229433354**	0.0011
					chr3:12815299	0.001
					**chr4:70722006**	0.0008
					chr9:22005510	0.0008
					chr1:1937724	0.0006
					chr1:229433453	0.0006
					chr4:70721924	0.0005
					**chr1:1937681**	0.0005
					chr4:70722010	0.0005
					chr1:1937706	0.0004
					chr1:1937771	0.0004
					chr1:1937599	0.0004
					chr1:1937577	0.0004
					chr19:10636497	0.0003
					chr7:157014861	0.0003
					chr1:1937621	0.0003
					chr3:44762007	0.0002
					chr3:12815440	0.0002
					chr3:12815404	0.0002
					chr11:71998932	0.0002
					chr1:1937587	0.0002
					chr1:1937608	0.0002
					chr19:57527261	0.0001
					chr7:157014845	0.0001
					chr11:71998905	0.0001
