# Supplementary material for: Epigenetic aging signatures and age prediction in human skeletal muscle
Source: Aging (Albany NY). 2025 Nov 26;17(11):2809–43. doi: 10.18632/aging.206341 (PMC12705185; doi:10.18632/aging.206341)
Supplement: Supplementary Tables 3-8 [file aging-17-11-206341-s004.pdf]

SUPPLEMENTARY TABLES

Supplementary Table 3. Summary of reduced CpG models: performance metrics under elastic net regularization and post-hoc thresholding.

Methods		Alpha regularization		Alpha regularization		Post-hoc thresholding	
Modeling		Elastic net; alpha = 0.5, l1_ratio = 0.9852		Elastic net; alpha = 0.8, l1_ratio = 0.9852		Elastic net; alpha = 0.0178, l1_ratio = 0.9852	
n of CpG		8		5		6	
train	MAE	9.377		11.509		5.052	
	RMSE	12.0175		14.4748		5.943	
	r	0.8109		0.7299		0.934	
	R-squared	0.4479		0.1991		0.865	
test	MAE	10.4848		11.9844		7.364	
	RMSE	12.7364		14.6885		11.3	
	r	0.7628		0.6897		0.726	
	R-squared	0.3742		0.1677		0.507	
Formula		Position	Coefficient	Position	Coefficient	Position	Coefficient
		intercept	55.5899595	intercept	44.53694062	intercept	163.3170259
		chr3:12815297	−6.566861612	chr19:10636467	−6.493581544	chr3:44761961	53.498989
		chr19:10636467	−15.12568411	chr9:22005565	8.777387791	chr17:29042762	38.623448
		chr3:12815285	−9.302902911	chr9:22005510	3.293509568	chr3:12815466	−30.351224
		chr3:12815282	−2.1355773	chr9:22005577	5.634173089	chr3:44761965	29.531605
		chr9:22005565	13.39037812	chr7:143316521	−2.470404842	chr19:57527206	30.667327
		chr9:22005510	6.784154861			chr3:12815426	−40.055187
		chr9:22005577	10.73001316				
		chr7:143316521	−9.800951957				

Supplementary Table 4. Hallmarks of Aging and functional annotations of the 20 skeletal muscle–specific CpG markers.

Marker	CpG_ID	RefGene	Hallmark of aging	Functional annotation	Supplementary References
MA_01	cg06458239	*ZNF549*	Epigenetic Alteration	Zinc finger transcription factor	[1, 2]
MA_02	cg02426178	*SLC44A2*	Mitochondrial Dysfunction	Choline transporter, Mitochondrial choline transporter regulating ATP production, oxidative stress, and metabolic homeostasis.	[3, 4]
MA_03	cg11456906	*CFAP74*	Altered Intercellular Communication	Ciliary and flagellar motility	[5]
MA_04	cg07743451	*TPM3*	Mitochondrial Dysfunction	Thin filament stabilization (actin-binding)	[6, 7]
MA_05	cg14812508	*TWF2*	Cytoskeletal Integrity	Actin-binding protein involved in cell motility	[8]
MA_06	cg07188513	*MNX1-AS1*	.	.	
MA_07	cg07502461	*TWF2*	Cytoskeletal Integrity	Actin-binding protein involved in cell motility	[8]
MA_08	cg09926178	*ACTA1*	Cytoskeletal Integrity	Muscle contraction regulation	[9, 10]
MA_09	cg15730967	*CAND2*	Altered Intercellular Communication	Heart muscle development, cAMP signaling pathway	[11]

MA_10	cg07303143	*KIF15*	Stem Cell Exhaustion	Mitotic spindle and proliferation regulation	[12]
MA_11	cg17722263	*MYCNUT*	Proteostasis Loss	Ubiquitin−proteasome system and protein degradation	[13]
MA_12	cg05543030	.	.	.	
MA_13	cg08390209	*CDKN2B*	Cellular Senescence	Cell cycle arrest via INK4 family inhibition	[14–17]
MA_14	cg19145398	*FOXS1*	Epigenetic Alteration	Transcriptional regulation via Forkhead box pathway	[18]
MA_15	cg26149678	*IL18BP*	Inflammaging	Immune signaling modulation, antagonist of IL-18	[19]
MA_16	cg12966875	*SLPI*	Inflammaging	Protease inhibitor, innate immune response modulator	[20]
MA_17	cg06059810	*RUFY3*	Altered Intercellular Communication	Vesicle trafficking and autophagy regulation	[21, 22]
MA_18	cg17922226	*CLCN1*	Neuromuscular Junction Dysfunction	Chloride ion channel in skeletal muscle excitability	[23]
MA_19	cg06144905	*PIPOX*	Mitochondrial Dysfunction	Peroxisomal amino acid and redox metabolism	[24]
MA_20	cg13985639	*DHDPSL*	Mitochondrial Dysfunction	Amino acid metabolism, mitochondrial enzymatic function	[25]

Supplementary Table 5. Primers for quantitative real-time reverse transcription PCR (qRT-PCR).

Related CpG	RefGene	Forward primer sequence (5′→3′)	Reverse primer sequence (5′→3′)	Size (bp)
cg06458239	*ZNF549*	CTTTACCGCCCGCCTTTC	GGCACCTTTGAGCTTCATCA	155
cg02426178	*SLC44A2*	TCATGTTCTTCTGGTTGGCC	ATCTGCACAATGGCCAGGAT	199
cg11456906	*CFAP74*	AAGGCCGAGGAACACAGATT	GCGGCAAACTTGATCTGGTA	128
cg07743451	*TPM3*	GAAGTTGAGGGAGAAAGGCG	CTCCTGAGCACGGTCCAG	105
cg14812508	*TWF2*	CCTGGTCGCCTGATAACTCC	ACAGGACGACAGGTGTTTCT	164
cg07188513	*MNX1-AS1*	CAAAGCTCTGCAGGTCGAAC	TGCATGTGTTTGGTGGCTAC	113
cg09926178	*ACTA1*	GGACAGCGCCAAGTGAAG	CGTCTTCGTCGCACATTGT	111
cg15730967	*CAND2*	AGGATAGTGAATTCAGTGAGCAA	AGCCTTGACGTTCTCCTCG	200
cg07303143	*KIF15*	GGCTGCATTGTTTTCGGGAT	TCTTTCTGCAGGAGGACGAA	154
cg17722263	*MYCNUT*	CCGATGTACAGGCAGAACTTG	GACCACAGAACTCAGCCAGA	245
cg08390209	*CDKN2B*	CTGGAACCTAGATCGCCGAT	GGTGAGAGTGGCAGGGTC	140
cg19145398	*FOXS1*	GAAGCTGAGCCTGACCCA	CTTGGTTGGCTCAGTTGTGG	104
cg26149678	*IL18BP*	AAGGAAGGCTCTTCAGGACC	AGGAGCAGGACCCACAAAG	147
cg12966875	*SLPI*	AGAGTCACTCCTGCCTTCAC	TGGGCAGATTTCTTAGGAGGA	145
cg06059810	*RUFY3*	CTCACCGCTGCCCTTCCT	CAGCTTGGCCATGTTCATGA	127
cg17922226	*CLCN1*	GGACTGCCCTCTGAGAATGG	CCTATGTCCTGCTCCCTGTC	130
cg06144905	*PIPOX*	GCCTGTCTTTGCTTGCCTTT	CCTCGGGAGTGTGGTAGAAA	195
cg13985639	*DHDPSL*	GGGAAGAAGGTGGACATTGC	TGCTGGTCAGGAAAGGAAAC	172
	GAPDH	CCACTCCTCCACCTTTGACG	CCACCACCCTGTTGCTGTAG	211

Supplementary Table 6. Primers used for PCR with bisulfite-converted DNA as the template.

Marker	CpG_ID	Forward primer sequence (5′→3′)	Reverse primer sequence (5′→3′)	Size (bp)
MA_01	cg06458239	AGGTGTTTTTTTTTTGGGTAATGAT	CCTCTCTTTCTTAAATTAAATCCTCAC	129
MA_02	cg02426178	AGGGTTTAGTAGTAGGAGGTAAAG	ACCCTACTAAACCTACAAATCTTCA	116
MA_03	cg11456906	AGGTATAGAGGAATTTAGGGTAGAA	CTACACCTCCCTAAACCTAAAAAC	308
MA_04	cg07743451	GGGAGAGGTAGATAGTTTTT	CTATACTCACAAATACATTACCC	154
MA_05	cg14812508	GTTTAAAGTAGGGTGTGGTT	CCCCTACAAAATAACCTCT	208
MA_06	cg07188513	AGTTATTTTTTGTGTTAGGGGTAAG	CCCAAACACATTAATCCTATCCTAC	181
MA_07	cg07502461	TTGTTTTGGGGGTAGAGGT	ACAACTTTATACCTTCCCTTACT	197
MA_08	cg09926178	GAATTAGGAAAAGGGGTATAGG	CCCAACAAACAAACAATAC	207
MA_09	cg15730967	AGTGGTTGGGTAGGTAAAT	CACTACACCCTAACACCTATAC	286
MA_10	cg07303143	GGATATTGAAGGAAGGGATGAGTTT	CCCTATTTTTACCCCCAAATACAAC	176
MA_11	cg17722263	GAGATTAGGGGAAAGGTT	CCATCACTATCTTTAATCATTC	101
MA_12	cg05543030	TGTTGTTTTTGGGAGAGTTATTG	AAACTTTCAATATCTAAATCCCTATCT	222
MA_13	cg08390209	GAGTGGGAGAAGGTAGTGATTA	CCTCCACTTTATCCTCAATCTT	142
MA_14	cg19145398	TTGGGGAGGGATAGGATGTG	CTCCCTCTTTCTCCCTCTTATAAT	144
MA_15	cg26149678	GGTTGGTTTTYGAGTTTGTGTGTTAGT	CACTAATAATCCCAAACTCCTACCTA	211
MA_16	cg12966875	AGGTATAGAATAGGTATTGGGGATA	ACCAAACACAAACTCCCTACTA	214
MA_17	cg06059810	GTTGGGATGTTGAGTTTTTATAGGA	CTCCCAACATCAACAACATCTATA	168
MA_18	cg17922226	GTGAAAATGAGGAATTGGGTGAAAAGA	ACATACTTAAATAAACCCAAACTTTCAA	124
MA_19	cg06144905	GGAGGTGGGTTTTATTTTGG	CCTACTAATTTTCCCCTCTTC	95
MA_20	cg13985639	AGGGGAGATTGGTTTGGAGTTA	CAACCACATACCTTCACATCTACA	211

Supplementary Table 7. Four sets of primers used for single base extension (SBE).

Group	Marker	SBE primer sequence (5′→3′)	Size (bp)
**G1**	MA_15	$(T)_{50}$ TCCTCCTTATCTATAAAACTCTCAC	75
	MA_07	$(T)_{41}$ AAACTAAAAATCTACAACRAAAAAC	66
	MA_17	$(T)_{32}$ TTTTAATCAACACCCTACTTACTAC	57
	MA_13	$(T)_{23}$ CTCCTCAACAAACATTAAAATAAAC	48
	MA_01	$(T)_{14}$ CTTTCTTAAATTAAATCCTCACAAC	39
	MA_02	$(T)_{5}$ CATATTCTTCTAATTAACCAACTTC	30
**G2**	MA_09	$(T)_{50}$ ATACTCATCCRCCRCTTCAAAAAAC	75
	MA_20	$(T)_{41}$ TAACAAAATTAACAAAAACTTTCAC	66
	MA_10	$(T)_{32}$ TATAAAAAAATAACAACCTCRAACC	57
	MA_14	$(T)_{23}$ CTCATTTTATTTTTATCTCTATCCC	48
	MA_18	$(T)_{14}$ TTTCATATTTTAAACACATCACCAC	39
	MA_19	$(T)_{5}$ TACTTTAAAAATTCTAAAAAACCCC	30
**G3**	MA_06	$(T)_{45}$ AATCTTCRAAAACTCATACAATTCC	70
	MA_08	$(T)_{35}$ ACAAAAAACTTCTCAATAAAATCTC	60
	MA_05	$(T)_{25}$ TTACTAAATACCAAAAACACCTATC	50
	MA_16	$(T)_{15}$ CTTAATTCCCTAAAATATTTACACC	40
	MA_12	$(T)_{5}$ CTCAAAAAACTTAAATTAAAACCC	29
**G4**	MA_11	$(T)_{35}$ TTCAAAATACAACCRCATACRCTAC	60
	MA_04	$(T)_{20}$ CAAATACATTACCCAAAAAAATACC	45
	MA_03	$(T)_{5}$ TAAATTCTTATTACTACTAAAACC	29

Supplementary Table 8. Primers for single multiplexes of markers in the highest performance model for SBE.

Marker	SBE Primer Sequence (5′→3′)	Size (bp)
MA_19	$(T)_{1}$ TACTTTAAAAATTCTAAAAAACCCC	26
MA_13	$(T)_{10}$ CTCCTCAACAAACATTAAAATAAAC	35
MA_04	$(T)_{20}$ CAAATACATTACCCAAAAAAATACC	45
MA_01	$(T)_{30}$ CTTTCTTAAATTAAATCCTCACAAC	55
MA_08	$(T)_{41}$ ACAAAAAACTTCTCAATAAAATCTC	66
MA_18	$(T)_{52}$ TTTCATATTTTAAACACATCACCAC	77
MA_10	$(T)_{62}$ TATAAAAAAATAACAACCTCRAACC	87

SUPPLEMENTARY REFERENCES

1. Cassandri M, Smirnov A, Novelli F, Pitolli C, Agostini M, Malewicz M, Melino G, Raschellà G. Zinc-finger proteins in health and disease. Cell Death Discov. 2017; 3:17071. https://doi.org/10.1038/cddiscovery.2017.71 PMID:29152378

2. Kamaliyan Z, Clarke TL. Zinc finger proteins: guardians of genome stability. Front Cell Dev Biol. 2024; 12:1448789. https://doi.org/10.3389/fcell.2024.1448789 PMID:39119040

3. Traiffort E, O'Regan S, Ruat M. The choline transporter-like family SLC44: properties and roles in human diseases. Mol Aspects Med. 2013; 34:646–54. https://doi.org/10.1016/j.mam.2012.10.011 PMID:23506897

4. Bennett JA, Mastrangelo MA, Ture SK, Smith CO, Loelius SG, Berg RA, Shi X, Burke RM, Spinelli SL, Cameron SJ, Carey TE, Brookes PS, Gerszten RE, et al. The choline transporter Slc44a2 controls platelet activation and thrombosis by regulating mitochondrial function. Nat Commun. 2020; 11:3479. https://doi.org/10.1038/s41467-020-17254-w PMID:32661250

5. Zhang X, Huang G, Jiang T, Meng L, Li T, Zhang G, Wu N, Chen X, Zhao B, Li N, Wu S, Guo J, Zheng R, et al. CEP112 coordinates translational regulation of essential fertility genes during spermiogenesis through phase separation in humans and mice. Nat Commun. 2024; 15:8465. https://doi.org/10.1038/s41467-024-52705-8 PMID:39349455

6. Matyushenko AM, Nefedova VV, Shchepkin DV, Kopylova GV, Berg VY, Pivovarova AV, Kleymenov SY, Bershitsky SY, Levitsky DI. Mechanisms of disturbance of the contractile function of slow skeletal muscles induced by myopathic mutations in the tropomyosin TPM3 gene. FASEB J. 2020; 34:13507–20. https://doi.org/10.1096/fj.202001318R PMID:32797717

7. Lambert MR, Gussoni E. Tropomyosin 3 (TPM3) function in skeletal muscle and in myopathy. Skelet Muscle. 2023; 13:18. https://doi.org/10.1186/s13395-023-00327-x PMID:37936227

8. Nevalainen EM, Skwarek-Maruszewska A, Braun A, Moser M, Lappalainen P. Two biochemically distinct and tissue-specific twinfilin isoforms are generated from the mouse Twf2 gene by alternative promoter usage. Biochem J. 2009; 417:593–600. https://doi.org/10.1042/BJ20080608 PMID:18837697

9. Labasse C, Brochier G, Taratuto AL, Cadot B, Rendu J, Monges S, Biancalana V, Quijano-Roy S, Bui MT, Chanut A, Madelaine A, Lacène E, Beuvin M, et al. Severe ACTA1-related nemaline myopathy: intranuclear rods, cytoplasmic bodies, and enlarged perinuclear space as characteristic pathological features on muscle biopsies. Acta Neuropathol Commun. 2022; 10:101. https://doi.org/10.1186/s40478-022-01400-0 PMID:35810298

10. Clayton JS, Johari M, Taylor RL, Dofash L, Allan G, Monahan G, Houweling PJ, Ravenscroft G, Laing NG. An Update on Reported Variants in the Skeletal Muscle *α*-Actin (*ACTA1*) Gene. Hum Mutat. 2024; 2024:6496088. https://doi.org/10.1155/2024/6496088 PMID:40225930

11. Górska AA, Sandmann C, Riechert E, Hofmann C, Malovrh E, Varma E, Kmietczyk V, Ölschläger J, Jürgensen L, Kamuf-Schenk V, Stroh C, Furkel J, Konstandin MH, et al. Muscle-specific Cand2 is translationally upregulated by mTORC1 and promotes adverse cardiac remodeling. EMBO Rep. 2021; 22:e52170. https://doi.org/10.15252/embr.202052170 PMID:34605609

12. Yin YX, Ding MQ, Yi Y, Zou YJ, Liao BY, Sun SC. Insufficient KIF15 during porcine oocyte ageing induces HDAC6-based microtubule instability. Theriogenology. 2024; 226:49–56. https://doi.org/10.1016/j.theriogenology.2024.05.043 PMID:38838614

13. Li S, Jiang C, Pan J, Wang X, Jin J, Zhao L, Pan W, Liao G, Cai X, Li X, Xiao J, Jiang J, Wang P. Regulation of c-Myc protein stability by proteasome activator REGγ. Cell Death Differ. 2015; 22:1000–11. https://doi.org/10.1038/cdd.2014.188 PMID:25412630

14. Li Y, Li C, Zhou Q, Liu X, Qiao Y, Xie T, Sun H, Ong MT, Wang H. Multiomics and cellular senescence profiling of aging human skeletal muscle uncovers Maraviroc as a senotherapeutic approach for sarcopenia. Nat Commun. 2025; 16:6207. https://doi.org/10.1038/s41467-025-61403-y PMID:40617829

15. Tumasian RA 3rd, Harish A, Kundu G, Yang JH, Ubaida-Mohien C, Gonzalez-Freire M, Kaileh M, Zukley LM, Chia CW, Lyashkov A, Wood WH 3rd, Piao Y, Coletta C, et al. Skeletal muscle transcriptome in healthy aging. Nat Commun. 2021; 12:2014. https://doi.org/10.1038/s41467-021-22168-2 PMID:33795677

16. Nanda V, Downing KP, Ye J, Xiao S, Kojima Y, Spin JM, DiRenzo D, Nead KT, Connolly AJ, Dandona S, Perisic

L, Hedin U, Maegdefessel L, et al. CDKN2B Regulates TGFβ Signaling and Smooth Muscle Cell Investment of Hypoxic Neovessels. Circ Res. 2016; 118:230–40. https://doi.org/10.1161/CIRCRESAHA.115.307906 PMID:26596284

17. Koch CM, Suschek CV, Lin Q, Bork S, Goergens M, Joussen S, Pallua N, Ho AD, Zenke M, Wagner W. Specific age-associated DNA methylation changes in human dermal fibroblasts. PLoS One. 2011; 6:e16679. https://doi.org/10.1371/journal.pone.0016679 PMID:21347436

18. Kuang Y, Yu Y, Wang C, Li H, Zhou Y, Pan L, Zhang Y, Cheng X, Jiang Z, Hu X. FOXS1, frequently inactivated by promoter methylation, inhibited colorectal cancer cell growth by promoting TGFBI degradation through autophagy-lysosome pathway. J Adv Res. 2025; 77:623–36. https://doi.org/10.1016/j.jare.2025.01.037 PMID:39864590

19. Zhang LM, Zhang Y, Fei C, Zhang J, Wang L, Yi ZW, Gao G. Neutralization of IL-18 by IL-18 binding protein ameliorates bleomycin-induced pulmonary fibrosis via inhibition of epithelial-mesenchymal transition. Biochem Biophys Res Commun. 2019; 508:660–6. https://doi.org/10.1016/j.bbrc.2018.11.129 PMID:30527805

20. Mongkolpathumrat P, Pikwong F, Phutiyothin C, Srisopar O, Chouyratchakarn W, Unnajak S, Nernpermpisooth N, Kumphune S. The secretory leukocyte protease inhibitor (SLPI) in pathophysiology of non-communicable diseases: Evidence from experimental studies to clinical applications. Heliyon. 2024; 10:e24550. https://doi.org/10.1016/j.heliyon.2024.e24550 PMID:38312697

21. Wei Z, Sun M, Liu X, Zhang J, Jin Y. Rufy3, a protein specifically expressed in neurons, interacts with actin-bundling protein Fascin to control the growth of axons. J Neurochem. 2014; 130:678–92. https://doi.org/10.1111/jnc.12740 PMID:24720729

22. Char R, Pierre P. The RUFYs, a Family of Effector Proteins Involved in Intracellular Trafficking and Cytoskeleton Dynamics. Front Cell Dev Biol. 2020; 8:779. https://doi.org/10.3389/fcell.2020.00779 PMID:32850870

23. Vivekanandam V, Jayaseelan D, Hanna MG. Muscle channelopathies. Handb Clin Neurol. 2023; 195:521–32. https://doi.org/10.1016/B978-0-323-98818-6.00006-6 PMID:37562884

24. Kim J, Bai H. Peroxisomal Stress Response and Inter-Organelle Communication in Cellular Homeostasis and Aging. Antioxidants (Basel). 2022; 11:192. https://doi.org/10.3390/antiox11020192 PMID:35204075

25. Belostotsky R, Seboun E, Idelson GH, Milliner DS, Becker-Cohen R, Rinat C, Monico CG, Feinstein S, Ben-Shalom E, Magen D, Weissman I, Charon C, Frishberg Y. Mutations in DHDPSL are responsible for primary hyperoxaluria type III. Am J Hum Genet. 2010; 87:392–9. https://doi.org/10.1016/j.ajhg.2010.07.023 PMID:20797690
